# Inhibiting the glycerophosphodiesterase EDI3 in ER-HER2+ breast cancer cells resistant to HER2-targeted therapy reduces viability and tumour growth

**DOI:** 10.1186/s13046-022-02578-w

**Published:** 2023-01-20

**Authors:** Magdalena Keller, Katharina Rohlf, Annika Glotzbach, Gregor Leonhardt, Simon Lüke, Katharina Derksen, Özlem Demirci, Defne Göçener, Mohammad AlWahsh, Jörg Lambert, Cecilia Lindskog, Marcus Schmidt, Walburgis Brenner, Matthias Baumann, Eldar Zent, Mia-Lisa Zischinsky, Birte Hellwig, Katrin Madjar, Jörg Rahnenführer, Nina Overbeck, Jörg Reinders, Cristina Cadenas, Jan G. Hengstler, Karolina Edlund, Rosemarie Marchan

**Affiliations:** 1 Leibniz Research Centre for Working Environment and Human Factors at the TU Dortmund (IfADo), Ardeystrasse 67, 44139 Dortmund, Germany; 2 Leibniz Institut Für Analytische Wissenschaften - ISAS E.V, Dortmund, Germany; 3 Institute of Pathology and Medical Research Center (ZMF), University Medical Center Mannheim, Heidelberg University, Mannheim, Germany; 4 Department of Pharmacy, AlZaytoonah University of Jordan, Amman, Jordan; 5 Department of Immunology Genetics and Pathology, Uppsala University, Uppsala, Sweden; 6 Department of Obstetrics and Gynecology, University Medical Center Mainz, Mainz, Germany; 7 Pharmacology Department, Lead Discovery Center, Dortmund, Germany; 8 Department of Statistics, TU Dortmund University, Dortmund, Germany

**Keywords:** Breast cancer, HER2 positive breast cancer, Choline metabolism, HER2-targeting therapy resistance, GPCPD1

## Abstract

**Background:**

Intrinsic or acquired resistance to HER2-targeted therapy is often a problem when small molecule tyrosine kinase inhibitors or antibodies are used to treat patients with HER2 positive breast cancer. Therefore, the identification of new targets and therapies for this patient group is warranted. Activated choline metabolism, characterized by elevated levels of choline-containing compounds, has been previously reported in breast cancer. The glycerophosphodiesterase EDI3 (*GPCPD1*), which hydrolyses glycerophosphocholine to choline and glycerol-3-phosphate, directly influences choline and phospholipid metabolism, and has been linked to cancer-relevant phenotypes in vitro. While the importance of choline metabolism has been addressed in breast cancer, the role of EDI3 in this cancer type has not been explored.

**Methods:**

EDI3 mRNA and protein expression in human breast cancer tissue were investigated using publicly-available Affymetrix gene expression microarray datasets (*n* = 540) and with immunohistochemistry on a tissue microarray (*n* = 265), respectively. A panel of breast cancer cell lines of different molecular subtypes were used to investigate expression and activity of EDI3 in vitro. To determine whether EDI3 expression is regulated by HER2 signalling, the effect of pharmacological inhibition and siRNA silencing of HER2, as well as the influence of inhibiting key components of signalling cascades downstream of HER2 were studied. Finally, the influence of silencing and pharmacologically inhibiting EDI3 on viability was investigated in vitro and on tumour growth in vivo.

**Results:**

In the present study, we show that EDI3 expression is highest in ER-HER2 + human breast tumours, and both expression and activity were also highest in ER-HER2 + breast cancer cell lines. Silencing HER2 using siRNA, as well as inhibiting HER2 signalling with lapatinib decreased EDI3 expression. Pathways downstream of PI3K/Akt/mTOR and GSK3β, and transcription factors, including HIF1α, CREB and STAT3 were identified as relevant in regulating EDI3 expression. Silencing EDI3 preferentially decreased cell viability in the ER-HER2 + cells. Furthermore, silencing or pharmacologically inhibiting EDI3 using dipyridamole in ER-HER2 + cells resistant to HER2-targeted therapy decreased cell viability in vitro and tumour growth in vivo.

**Conclusions:**

Our results indicate that EDI3 may be a potential novel therapeutic target in patients with HER2-targeted therapy-resistant ER-HER2 + breast cancer that should be further explored.

**Supplementary Information:**

The online version contains supplementary material available at 10.1186/s13046-022-02578-w.

## Background

Targeted therapies have proven tremendously effective to treat HER2 (*ERBB2*)-positive breast cancer. However, acquired or inherent resistance has limited the degree of success [1], thus highlighting the need for novel treatment options. Research that examines the suitability of targeting various proteins and processes regulating cellular metabolic capacity that are altered in cancer is ongoing [2]. Thus far, the focus has been on glucose, lipid, nucleotide, and amino acid metabolism [2]. Nevertheless, choline metabolism has also been reported to be deregulated in breast cancer [3–10]. Previously, we identified the glycerophosphodiesterase EDI3 (*GPCPD1*), which hydrolyses glycerophosphocholine (GPC) to produce choline and glycerol-3-phosphate (G3P), as a key player in choline metabolism, and showed that it is linked to metastasis and worse outcome in endometrial and ovarian carcinoma [11]. Our work also supported the role of EDI3 in cancer-relevant phenotypes, namely, cell migration, adhesion, and spreading [11, 12]. In addition, silencing EDI3 decreased levels of the signalling lipids lysophosphatidic acid and phosphatidic acid, as well as phosphatidylcholine, the most abundant phospholipid in cell membranes [11, 13, 14]. However, despite the available evidence linking choline metabolism and breast cancer, EDI3 has not yet been extensively studied in this cancer type. Thus, we investigated EDI3’s potential clinical relevance using publicly available data, as well as our own breast cancer patient cohorts, and show that EDI3 expression is highest in estrogen receptor (*ESR1*)-negative (ER-) HER2 + tumours. Our subsequent experiments indicate that EDI3 is regulated by HER2 signalling in ER-HER2 + cell lines, leading to the hypothesis that targeting EDI3 could be a therapeutic approach to enhance the impact of standard therapies or an alternative in case of resistance to standard therapies, which we evaluated using in vitro and in vivo models.

## Material and methods

### Human breast cancer datasets

EDI3 expression in human breast cancer tissue was evaluated using publicly-available Affymetrix gene expression microarray datasets analysed on the Affymetrix HG-U133-Plus 2.0 array (Supplementary Table S1). The CEL files were downloaded from the Gene Expression Omnibus (GEO) (https://www.ncbi.nlm.nih.gov/geo/) and normalized using frozen Robust Multiarray Analysis [15].

### Immunohistochemistry

**Fig. 1:**
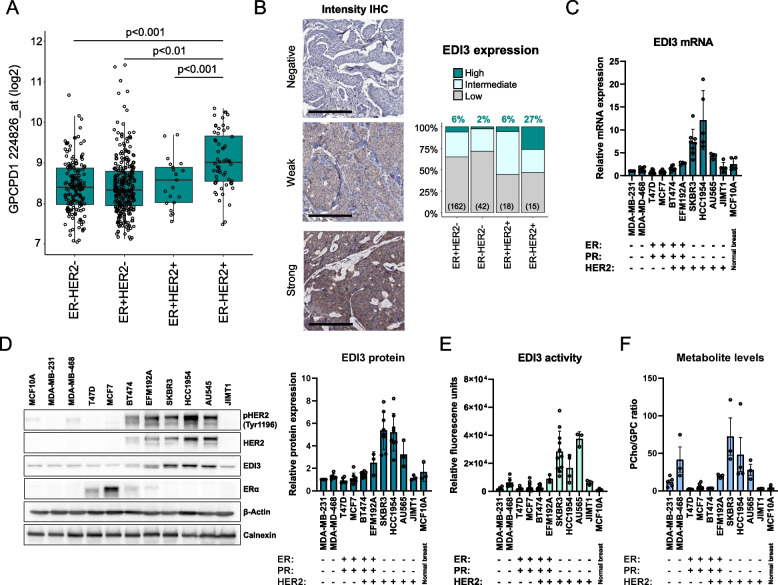
EDI3 expression and enzyme activity according to HER2 and ER status. **A,** EDI3 (*GPCPD1*; 224826_at) mRNA expression was compared among subtypes defined according to HER2 and ER status in human breast cancer Affymetrix datasets (total *n* = 540). **B,** EDI3 protein expression in breast cancer subtypes using IHC on a TMA (*n* = 265); high expression was defined as strong positivity in > 50% of tumour cells, intermediate as weak in >50% or strong in ≤ 50% of tumour cells, and low as negative or weak in ≤ 50% of tumour cells. **C,** qPCR analysis of EDI3 mRNA expression in a panel of breast cancer cell lines of different molecular subtypes normalized to β-Actin. **D,** Representative Western blot (left panel) showing pHER2, HER2, EDI3 and ERα protein expression in the panel of breast cancer cell lines, with quantification normalized to β-Actin (right panel). **E,** EDI3 enzyme activity measured using an enzyme-coupled assay that indirectly measures the release of choline. **F,** Intracellular PCho/GPC ratio measured using NMR spectroscopy. Cell line data (**C-F**) are mean ± SD of at least three independent experiments. IHC, immunohistochemistry; TMA, tissue microarray; NMR, nuclear magnetic resonance

EDI3 and HER2 protein expression were assessed by immunohistochemistry (IHC) on a tissue microarray (TMA), including 265 human breast cancers, from patients who were operated on at the Department of Obstetrics and Gynecology, University Medical Center Mainz, Germany between 1988 and 2000. A custom-made mouse monoclonal antibody (clone 3B8G3, AMS Bio) raised against full-length recombinant EDI3 protein produced in HEK293 cells (TP305217, Origene) and the anti-HER2 mouse monoclonal antibody AMAb90627 (Atlas Antibodies) were used. IHC conditions are described in the Supplementary Methods. The stained slides were scanned using an Aperio AT2 whole-slide scanner (Leica Biosystems) and the resulting high-resolution images were manually annotated. Cytoplasmic EDI3 positivity in the tumour cells was considered. The staining intensity was scored semi-quantitatively as negative, weak, or strong (Fig. 1B, left panel). The estimated fraction of positive cells was scored as 0–1%, 2–25%, 26–50%, 51–75% or > 75%. Intensity and fraction were then combined into a total EDI3 expression score as follows: [1] low expression (negative or weak in ≤ 50%), [2] intermediate expression (weak in > 50% or strong in ≤ 50%), or [3] high expression (strong in > 50%) (Fig. 1B, right panel).

### Cell lines and cell culture

Human breast cancer cell lines MDA-MB-231, MDA-MB-468, MCF7, T47D, BT474, EFM192A, SKBR3, and JIMT1 were purchased from the German Collection of Microorganisms and Cell Cultures (DSMZ), and HCC1954, AU565, and MCF10A from the American Type Culture Collection (ATCC). The MCF7/NeuT cell line, with doxycycline-inducible expression of oncogenic HER2, was used as previously described [16]. All cell lines were authenticated by DSMZ according to the ANSI/ATCC ASN-0002–2011 guidelines, and were regularly tested for mycoplasma using the Venor® GeM Classic kit (Minerva Biolabs). Culture conditions for all lines are provided in the Supplementary Methods.

### Expression and activity analyses

Total RNA was isolated using the RNeasy Mini Kit (Qiagen), according to the manufacturer’s instructions, and quantified using NanoDrop 2000. Two μg of total RNA was transcribed into cDNA with the High-Capacity cDNA Reverse Transcription Kit (Qiagen), and qRT-PCR performed with QuantiFast SYBR Green (Qiagen) with QuantiTect primer assays (Supplementary Table S2A) on the ABI 7500 Fast Real-Time PCR system (Applied Biosystems). The relative expression of each target gene was calculated using 2^−ΔΔCT^.

For protein expression analysis, cells were lysed in RIPA buffer containing phosphatase and protease inhibitors (Sigma Aldrich), and the total protein concentration was measured using the BCA Protein Assay Kit (Thermo Fisher). Equal amounts of protein were separated on SDS polyacrylamide gels, transferred onto PVDF membranes, and protein expression was detected using antibodies listed in Supplementary Table S2B. Images were taken on a Fusion Fx7 imager (Vilber) and bands quantified using ImageJ (National Institute of Health). In addition, EDI3 and HER2 protein expression was analysed with immunocytochemistry, as described in Supplementary Methods.

EDI3 activity was measured employing a modified version of the Amplex® Red Phospholipase D Assay Kit (Thermo Fisher Scientific), as previously described [11].

### Analysis of choline metabolites

Intracellular concentrations of GPC, choline, and PCho were measured using nuclear magnetic resonance (NMR) spectroscopy at 14.1 T, as described in the Supplementary Methods.

### Altering EDI3 and HER2 expression

EDI3 and HER2 expression were transiently downregulated in SKBR3, HCC1954, BT474, and EFM192A cells using siRNA and reverse transfection with Lipofectamine RNAiMax Reagent (Thermo Fisher) according to the manufacturer’s instructions. siRNA oligos targeting different exons of EDI3 and HER2 were used to silence gene expression, and non-targeting siRNAs were used as negative controls (all from Thermo Fisher Scientific). siRNA sequences are listed in Supplementary Table S2C, and transfection conditions are detailed in the Supplementary Methods. To induce HER2 expression, MCF7/NeuT cells were seeded overnight, followed by treatment with 1 µg/ml doxycycline (Sigma-Aldrich) for 24 h, 72 h or 7d. NeuT expression efficiency was monitored by an increase in the fluorescence of co-expressed EGFP. To stably and inducibly silence EDI3, HCC1954 cells were transduced with the SMARTvector™ lentiviral particles (Dharmacon) containing three shRNA oligos targeting different exons of EDI3 (Supplementary Table S2C) under the control of a Tet-On 3G tetracycline-inducible system, as well as a non-targeting scrambled shRNA control. Two days post-transduction, cells were incubated in selection media containing 0.5 µg/ml puromycin for two weeks to select the cells in which the vector was stably integrated. Dose and time dependent analyses were performed to determine the optimal concentration of doxycycline needed to specifically decrease EDI3 expression.

### Cell viability

Seventy two h after siRNA transfection, cells were re-seeded onto 96-well plates and treated with trastuzumab or lapatinib and respective vehicle controls for 96 h, with media changed after 48 h. Alternatively, cells were seeded onto 96-well plates and allowed to attach for 6 h, followed by treatment with 25 µM or 50 µM dipyridamole (Sigma) and 0.01, or 0.1 µM lapatinib (Selleckchem) for 96 h with media changed after 48 h. The percentage of viable cells was determined using the CellTiter-Blue® Cell Viability Assay (Promega), according to the manufacturer’s instructions, and fluorescence detected using a microplate reader (Tecan) with a 560Ex/590Em filter set.

### Colony formation assay

shRNA expression was induced with 0.01 and 0.1 µg/ml doxycycline in HCC1954 shEDI3 and control cells (shNEG). After 72h of doxycycline treatment, 500 cells were re-plated per well of a 6-well plate in doxycycline-containing media and incubated for 14d at 37 °C. Colonies were stained with crystal violet solution, photographed and the number and size of colonies analysed using ImageJ (National Institutes of Health).

### Proliferation assay

To investigate the effect of EDI3 silencing on proliferation, the Click-iT® Plus 5-Ethynyl-2-deoxyuridine (EdU) cell proliferation kit (Thermo Fisher Scientific) was used following the manufacturer’s instructions. Briefly, after 72 h doxycycline treatment, 1500 HCC1954 shNEG and HCC1954 shEDI3 cells were re-plated into 8-well chamber slides (Ibidi), allowed to attach overnight, and then incubated with 10 µM EdU for 2 h. The cells were then fixed with 4% paraformaldehyde for 15 min, permeabilized with 0.5% Triton®X-100 for 20 min, and then treated with the Click-iT® reaction cocktail with Alexa Fluor™ 488 dye for 30 min. DNA was stained with Hoechst 33342 for 30 min protected from light, followed by mounting with Fluoroshield mounting medium (Abcam). Fluorescent images were taken with a BZ-X800 microscope (Keyence) and the number of green fluorescent cells counted and normalized to total cell number and the respective 0 µg/ml doxycycline control cells.

### EDI3 expression studies after treatment with different pathway inhibitors

MCF7/NeuT cells were seeded onto a 6-well plate and allowed to attach overnight. On the next day, cells were treated with different concentrations of LY294002 (Selleckchem), PD98059 (Selleckchem), U73122 (Tocris), everolimus (Selleckchem) or CHIR-99021 (Selleckchem). For induction of the oncogenic rat HER2 variant, NeuT in the MCF7 cells, doxycycline (Sigma) was added with a final concentration of 1 µg/ml per well to one half of the experiment setup one hour after the inhibitor addition [16]. Medium was changed every three days, and cells were harvested after three and seven days, for RNA and protein expression analyses as described above.

For the EDI3 expression studies in endogenous HER2 expressing cell lines, SKBR3 and HCC1954 cells were seeded onto a 6-well plate and allowed to attach overnight. On the next day, cells were treated with different concentrations of lapatinib, everolimus, CHIR-99021, compound 3i (666–15), C188-9, KC7F2, SC75741, 10074-G5 or SR18662 (all from Selleckchem). After one, two and three days (as well as 4 days for lapatinib), cells were harvested at approximately 80% confluency for RNA and protein expression analyses as described above. Medium was changed after two days.

### Animal experiments

5 × 10^6^ HCC1954 cells (150 µl 1:1 Matrigel/1 × PBS) were injected subcutaneously into the flanks of 24 six- to eight-week-old female CD1 nude mice (Charles River). Tumour growth was measured 2 × weekly with a digital caliper and the volume calculated using the formula (L × W^2^)/2 [17]. Once the tumour volume was approximately 300 mm^3^, mice were randomly divided into four groups of six mice each and treated orally with vehicle, 120 mg/kg dipyridamole, 100 mg/kg lapatinib, or a combination of lapatinib and dipyridamole for 4 weeks, 5 days on/ 2 days off. Tumour size was monitored 2–3 times weekly, and mice were weighed daily. One hour after final treatment, blood and tumours were collected to analyse dipyridamole and lapatinib concentrations, as described in the Supplementary Methods.

HCC1954 shNEG and HCC1954 shEDI3 cells were treated with 0.1 μg/ml doxycycline 72 h prior to injection. 5 × 10^6^ HCC1954 shNEG or HCC1954 shEDI3 cells with and without doxycycline treatment (150 µl 1:1 Cultrex BME/1 × PBS) were injected subcutaneously into the flanks of seven, six- to eight-week-old female CD1 nude mice (Charles River) per condition. Mice with doxycycline induced cells were fed a diet containing 625 mg/kg doxycycline (Ssniff) ad libitum. Tumour growth was measured 2 × weekly with a digital caliper and the volume calculated using the formula (L × W^2^)/2 [17]. After 8 weeks tumours were collected, weighed, and analysed for RNA and protein analysis.

### Statistics

The Mann–Whitney U test or Kruskal Wallis test, as appropriate, was used to compare EDI3 expression between patient subgroups defined based on clinicopathological factors, categorized as: age: < 50 vs. ≥ 50 years; grade: I + II vs. III; pT stage: I vs. II + III; ER status: negative vs. positive; HER2 status: negative vs. positive; molecular subtype: ER + HER2 + vs. ER + HER2- vs. ER-HER2- vs. ER-HER2 + . For the Affymetrix cohorts, ER and HER2 status were derived from the bimodally distributed mRNA levels of the corresponding genes, as described in the Supplementary Methods.

All experiments were performed at least three times. Numerical data are presented as mean ± standard deviation (SD) or standard error (SE), as indicated in the respective figure legends. In vitro data were analysed using a two-sided Student’s t-test. Tumour weight and volume were analysed using Mann–Whitney U and two-sided Student’s t-tests, respectively. The significance level was set at *P *≤ 0.05. Analyses were performed using the statistical programming language R version 4.0.3 (http://www.r-project.org/), Statistical Package for the Social Sciences (SPSS) (Version 23, IBM), or GraphPad Prism, version 9.

## Results

### *EDI3 expression is high in ER − HER2* + *breast tumours*

To characterize EDI3 in human breast cancer, we investigated *EDI3* mRNA levels in publicly available Affymetrix datasets. Higher expression of *EDI3* (224826_at) was observed in HER2 + tumours compared to HER2 − tumours in the combined analysis of all datasets (*P* < 0.001, Mann–Whitney U test), as well as in four datasets when analysed separately. The highest EDI3 expression was observed in the ER − HER2 + subtype (Fig. 1A; Supplementary Table S3). To investigate whether this also holds true at protein level, we stained a TMA using a monoclonal antibody against EDI3. Overall, most tumours were negative for EDI3 or weak cytoplasmic positivity was observed in a variable cell fraction; strong EDI3 positivity was only observed in a smaller subset. Considering both the staining intensity and the fraction of EDI3-positive tumour cells, high protein expression was observed in 6% of ER + HER2-, 2% of ER − HER2-, 6% of ER + HER2 + , and 27% of ER − HER2 + tumours (Fig. 1B). Although EDI3 is not strongly expressed in every ER − HER2 + tumour, this subtype contains the largest percentage of tumours with high EDI3 expression, thus supporting the findings at mRNA level.

### *ER − HER2* + *breast cancer cell lines have high EDI3 expression and activity*

Cell lines representing four different subtypes of human breast cancer were examined to determine whether HER2, as well as ER status, and EDI3 expression are similarly associated as observed in tumour tissue. In agreement with the observation in the tumours, EDI3 expression was highest in the ER − HER2 + cell lines HCC1954 and SKBR3, followed by AU565 (Fig. 1C-D). Elevated EDI3 in SKBR3 cells was also visualized by immunocytochemistry (Supplementary Fig. S1C). One exception was JIMT1, which expressed low levels of EDI3 (Fig. 1C-D). An explanation may be that although JIMT1 is categorized as ER-HER2 + , its HER2 levels are low compared to the other HER2 + lines (Fig. 1D and Supplementary Fig. S1A) due to its expression of wild-type PTEN [18], which is suggested to negatively regulate HER2 expression [19].

We next investigated whether the high EDI3 expression observed in ER-HER2 + cell lines is associated with increased enzymatic activity. Accordingly, EDI3 activity was highest in the ER − HER2 + cell lines, SKBR3, HCC1954 and AU565 (Fig. 1E). In comparison, activity remained comparably low in MDA-MB-231, T47D, MCF7, and BT474 cells, and was slightly elevated in MDA-MB-468, EFM192A, and JIMT1 (Fig. 1E). To understand the effects of EDI3 activity on choline metabolism, GPC, choline, and PCho levels were measured using NMR. Importantly, the PCho/GPC ratio, which was reported as higher in more aggressive compared to less aggressive breast cancer cell lines [9], was highest in SKBR3 and HCC1954 cells (Fig. 1F). Furthermore, in agreement with the observed high EDI3 activity, SKBR3 and HCC1954 were among the cell lines with the lowest levels of EDI3’s substrate GPC and the highest PCho levels (Supplementary Fig. S1D).

Overall, ER − HER2 + cell lines express high EDI3, which leads to higher enzymatic activity. These findings prompted us to more closely investigate the relationship between EDI3 and HER2.

### *EDI3 expression is regulated by HER2 signalling *via* GSK3β and mTOR*

**Fig. 2:**
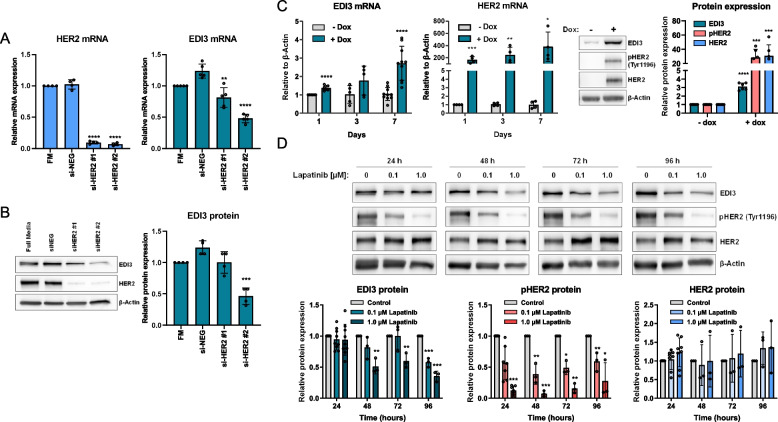
EDI3 expression is regulated by HER2 signalling. **A,** HER2 (left panel) and EDI3 (right panel) mRNA expression after silencing of HER2 in SKBR3 cells compared with cells transfected with scrambled siRNA (si-NEG). **B,** Protein expression of EDI3 and HER2 in SKBR3 cells after HER2 siRNA knockdown compared with control (si-NEG) (left panel) with accompanying quantification of EDI3 protein expression (right panel). **C,** qPCR analysis of EDI3 and HER2 mRNA expression in MCF-7/NeuT cells treated with doxycycline (+ dox) or untreated (− dox) at different time points (left panels) and representative Western blot showing expression of EDI3, pHER2 and HER2 in MCF-7/NeuT cells treated with doxycycline for 7 days, with corresponding quantification of EDI3 protein levels (right panels). **D,** Representative Western blots showing EDI3, pHER2 and HER2 protein expression in SKBR3 cells treated with 0.1 µM or 1.0 µM lapatinib for 24, 48, 72 and 96 h, with corresponding quantification of protein levels. Data are mean ± SD of at least three independent experiments (*, *P* < 0.05; **, *P* < 0.01; ***, *P* < 0.001; ****, *P* < 0.0001). FM, full media control

To understand the mechanism underlying high EDI3 expression in HER2 + breast cancer, we silenced HER2 using siRNA targeting different exons in ER − HER2 + cell lines. Silencing HER2 resulted in reduced EDI3 mRNA and protein expression in both SKBR3 (Fig. 2A-B) and HCC1954 (Supplementary Fig. S1E-F) cells. The reverse experiment showed that, despite successful downregulation of EDI3, HER2 expression and phosphorylation remained unchanged in both SKBR3 and HCC1954 cells (Supplementary Fig. S1G-J). We then used MCF7 breast cancer cells with doxycycline (dox)-inducible expression of NeuT [16], an oncogenic HER2/ERBB2 variant. Exposure of cells to dox caused the expected time-dependent increase in NeuT mRNA and protein expression (Fig. 2C). Notably, EDI3 expression also increased upon the addition of dox (Fig. 2C), further indicating that EDI3 expression is regulated by HER2. To confirm that signalling initiated upon the activation of HER2 is responsible for the high EDI3 expression, we treated cells with lapatinib, a reversible tyrosine kinase inhibitor. The selected lapatinib concentrations significantly inhibited HER2 phosphorylation in SKBR3 (Fig. 2D) and HCC1954 (Supplementary Fig. S1K) cells, and increased total HER2 expression, as previously reported [20]. Importantly, EDI3 expression significantly decreased in both cell lines (Fig. 2D; Supplementary Fig. S1K), albeit only at the highest lapatinib concentration after 96 h in HCC1954. This weak effect was not unexpected because HCC1954 cells contain a mutation in PIK3CA (H1047R) [18] that activates signalling downstream of HER2, making them less sensitive to inhibition of HER2 signalling.

**Fig. 3:**
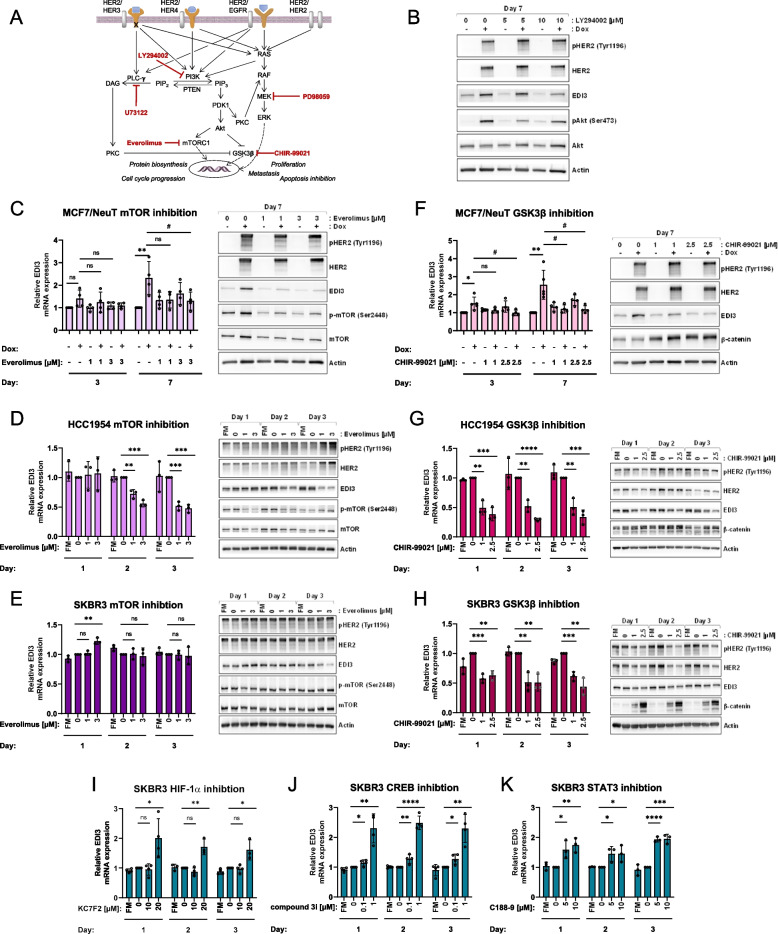
GSK3β and mTORC1 regulate EDI3 expression downstream of PI3K/Akt. **A,** Overview of key proteins in the HER2 pathway that initiate signalling upon HER2 activation, with the inhibitors used in the present study in red. **B,** Representative Western blot of pHER2, HER2, EDI3, pAkt, and total Akt protein levels in MCF-7/NeuT cells treated with 5 or 10 µM LY294002 for 7 days. **C,** mRNA expression of EDI3 (left) and representative Western blot of pHER2, HER2, EDI3, p-mTOR, and mTOR protein levels (right) in MCF-7/NeuT cells treated with 1 or 3 µM everolimus for 3 and 7 days. **D-E,** mRNA expression of EDI3 (left) and representative Western blot of pHER2, HER2, EDI3, p-mTOR, and mTOR protein levels (right) in **D,** HCC1954 and **E,** SKBR3 cells treated with 1 or 3 µM everolimus for 1, 2, and 3 days. **F,** mRNA expression of EDI3 (left) and representative Western blot of pHER2, HER2, EDI3, and β-catenin protein levels (right) in MCF-7/NeuT cells treated with 1 or 2.5 µM CHIR-99021 for 3 and 7 days. **G-H,** mRNA expression of EDI3 (left) and representative Western blot of pHER2, HER2, EDI3, and β-catenin protein levels (right) in **G,** HCC1954 and **H,** SKBR3 cells treated with 1 or 2.5 µM CHIR-99021 for 1, 2, and 3 days. **I-K,** EDI3 mRNA expression after inhibiting **I,** HIF1α with 10 and 20 µM KC7F2, **J,** CREB with 0.1 and 1 µM compound 3i , or **K,** STAT3 with 5 and 10 µM C188-9 for 1, 2 and 3 days in SKBR3 cells. Data represent the mean ± SD of at least three independent experiments (*, *P* < 0.05; **, *P* < 0.01; ***, *P* < 0.001; ****, *P* < 0.0001; ns, not significant). * in panels **C** and **F** indicate significant difference from untreated control; # represents difference to cells treated with dox alone

To determine which pathway downstream of HER2 regulates EDI3 expression, we inhibited key components of signalling cascades activated by HER2, namely phosphatidylinositol 3-kinase (PI3K; *PIK3CA*) with LY294002, phospholipase C gamma (PLCγ; *PLCG*) with U73122, and MEK1 (*MAP2K1*) with PD98059 (Fig. 3A) in MCF7-NeuT cells. Although not significant due to the variability in HER2 upregulation in response to dox, we observed reduced phosphorylation of Akt (*AKT1*), PKCα/βII (*PRKCA/PRKCB*), and ERK1/2 (*MAPK3*/*MAPK1*) in response to LY294002, U73122, and PD98059, respectively, compared with the dox control (Supplemental Fig. S2A-C). Only inhibition of PI3K with LY294002 led to a decrease in EDI3 expression as a result of dox-induced HER2 expression (Fig. 3B and Supplementary Fig. S2A), whereas targeting PLCγ and MEK1 had little to no effect (Supplementary Fig. S2B-C).

To investigate which pathway downstream of PI3K/Akt mediates EDI3 expression in ER − HER2 + cells, we next inhibited mechanistic target of rapamycin (mTOR) complex 1 (mTORC1; *AKT1S1*, *MLST8*, *MTOR*, *RPTOR*) and glycogen synthase kinase 3β (GSK3β; *GSK3B*) (Fig. 3A). Inhibiting mTORC1 with everolimus significantly decreased EDI3 mRNA and protein expression in MCF7-NeuT cells after 7 days dox treatment (Fig. 3C and Supplementary Fig. S2D). To confirm that this also holds true for endogenous EDI3, we treated both HCC1954 and SKBR3 cells with increasing concentrations of everolimus for 1, 2, and 3 days. EDI3 expression significantly decreased in a time- and concentration-dependent manner in HCC1954 cells (Fig. 3D and Supplementary Fig. S2E). Conversely, EDI3 expression remained predominantly unchanged in SKBR3 cells, with only a small decrease in protein levels observed after 3 days of treatment (Fig. 3E and Supplementary Fig. S2F). Indeed, SKBR3 cells have been reported as resistant to everolimus [21], which we also observed, as reduced phospho-mTOR (Ser4228) was seen only on day one in SKBR3 cells (Fig. 3E and Supplementary Fig. S2F), compared to the more consistent decrease in MCF7-NeuT and HCC1954 cells (Fig. 3C-D and Supplementary Fig. S2D-E).

Inhibiting GSK3β with CHIR-99021 resulted in an overall reduction in EDI3 mRNA and protein expression in dox-treated MCF7-NeuT cells (Fig. 3F and Supplementary Fig. S2G), which was significant at the higher concentration on both days. Moreover, CHIR-99021 also significantly decreased endogenous EDI3 expression in both HCC1954 and SKBR3 cells after 2 and 3 days of treatment for almost all conditions tested (Fig. 3G-H and Supplementary Fig. S2H-I). To investigate whether the selected concentrations of CHIR-99021 successfully inhibited GSK3β, we measured total β-catenin, which is normally phosphorylated by GSK3β, thus targeting it for ubiquitination and subsequent degradation. The selected CHIR-99021 concentrations led to an overall increase in total β-catenin (Fig. 3F-H and Supplementary Fig. S2G-I), which was higher in SKBR3 (Fig. 3H) than in MCF7-NeuT and HCC1954 cells (Fig. 3F-G), and may be attributed to the already high endogenous levels of total β-catenin in the latter two cell lines. Together, these data suggest that EDI3 expression is induced by HER2 signalling and regulated via factors downstream of PI3K/Akt, namely, mTORC1 and GSK3β.

### HIF1α, CREB and STAT3 control EDI3 expression downstream of GSK3β and mTORC1

To begin elucidating the transcriptional mechanism directly regulating EDI3 expression downstream of GSK3β and mTORC1, the promoter region of EDI3 was searched for potential transcription factor binding sites known to be regulated by mTORC1 or GSK3β using the transcription factor database JASPAR [22] and the MatInspector tool from Genomatix Software Suite [23]. The candidates identified included cAMP response element-binding protein (CREB), c-Myc, Krüppel-like factor 5 (KLF5), hypoxia-inducible factor 1a (HIF1α), signal transducer and activator of transcription 3 (STAT3), and nuclear factor kappa B (NFκB). To determine whether any of the identified transcription factors regulate EDI3 expression, ER-HER2 + SKBR3 cells were treated with small molecule inhibitors against each transcription factor, using two different concentrations, and EDI3 expression was measured over three days. Interestingly, none of the tested inhibitors significantly reduced EDI3 expression, and while no significant change in expression was observed upon inhibition of c-Myc with 10074-G5 (Fig. S2J), targeting CREB, STAT3 and HIF1α with compound 3i (666–15), C188-9 and KC7F2, respectively, led to significantly elevated EDI3 expression for all three days for at least one concentration tested compared to the vehicle control (Fig. 3I-K). We also observed significantly higher expression of EDI3 upon inhibition of NFκB and KLF5, with SC75741 and SR18662, respectively, but on days 1 and 2 for the former and only on day 3 for the latter (Fig. S2K-L). These data altogether suggest that EDI3 is negatively regulated by transcription factors downstream of GSK3β. Indeed, previous studies have shown that phosphorylation of CREB and HIF1α by GSK3β leads to their inactivation [24, 25]. Furthermore, phosphorylation of KLF5 by GSK3β targets it for proteasomal degradation [26]. Thus, inhibiting GSK3β would result in active CREB, HIF1α and KLF5 that are then free to bind the promoter of target genes to regulate expression. Similarly, an earlier study showed that inhibiting GSK3β increased STAT3 and NFκB expression [27], which, based on our findings, will inhibit EDI3 transcription. While more studies are needed to fully clarify the mechanism underlying EDI3’s regulation via GSK3β and mTORC1, these initial results present potential regulatory candidates that are themselves deregulated in breast cancer [28–31].

### *Loss of viability upon EDI3 silencing is more pronounced in ER − HER2* + *cells*

**Fig. 4:**
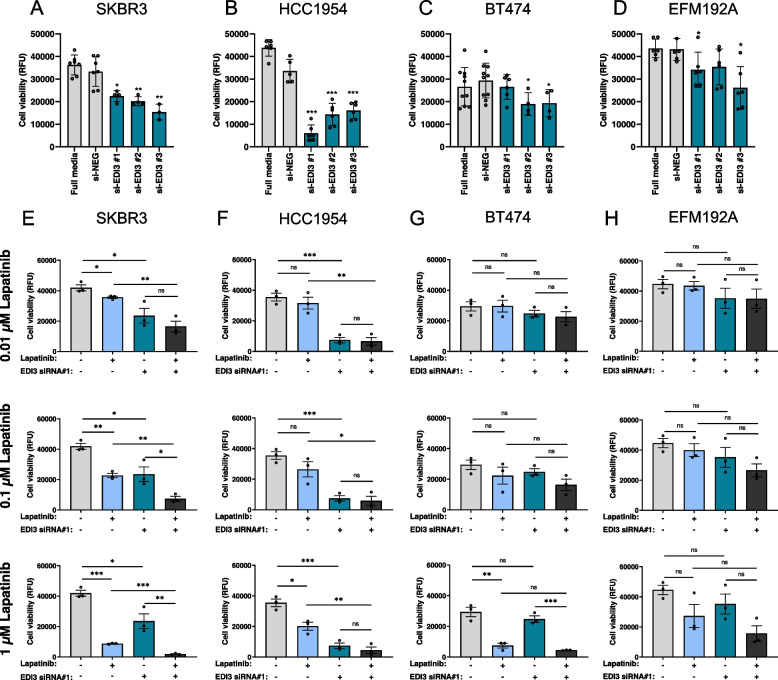
Cell viability and sensitivity to HER2-targeting treatment after silencing EDI3 using siRNA. Viability relative to negative control (si-NEG) after silencing EDI3 with three siRNAs targeting different exons in **A,** SKBR3. **B,** HCC1954, **C,** BT474 and **D,** EFM192A cells. Viability presented as RFU after silencing EDI3 using siRNA (oligo #1) with and without lapatinib (0.01, 0.1 and 1 µM) for 96 h in **E,** SKBR3, **F,** HCC1954, **G,** BT474 and **H,** EFM192 cells. Data are mean ± SD (A-D) and mean ± SE (E–H) of at least three independent experiments (*, *P* < 0.05; **, *P* < 0.01; ***, *P* < 0.001; ns, not significant). RFU, relative fluorescence units

Since EDI3 expression is regulated by proteins downstream of PI3K/Akt, which are closely linked to tumour development and progression, we next investigated whether the high EDI3 expression in the ER − HER2 + subtype is associated with improved viability, making it a potential therapeutic target. Our data show that silencing EDI3 resulted in a significant reduction in cell viability in both SKBR3 and HCC1954 cell lines (Fig. 4A-B). Conversely, silencing EDI3 in the ER + HER2 + cell lines BT474 and EFM192A (Supplementary Fig. S3A-B) had little to no effect (Fig. 4C-D). We then examined whether silencing EDI3 altered the sensitivity of these cells to HER2-targeted therapy. Cells transfected with EDI3 siRNA or non-targeting scrambled oligos were treated with different concentrations of lapatinib. Lapatinib alone resulted in a dose-dependent decrease in cell viability in all four cell lines (Supplementary Fig. S3C). As previously reported [18], SKBR3 (Fig. 4E and Supplementary Fig. S3D) and BT474 (Fig. 4G and Supplementary Fig. S3F) cells were sensitive to lapatinib, with a significant decrease in viability observed at all tested concentrations in SKBR3 cells. Only the highest concentration of lapatinib resulted in a marked reduction in viability in the less responsive [18] HCC1954 (Fig. 4F and Supplementary Fig. S3E), and EFM192A (Fig. 4H and Supplementary Fig. S3G) cell lines, which did not reach significance in the latter.

Combined EDI3 knockdown and inhibition of HER2 had a greater effect on cell viability than lapatinib alone in ER − HER2 + cells, even at the lowest lapatinib concentrations used (Fig. 4E-F and Supplementary Fig. S3D-E). This was not observed in the ER + HER2 + cells (Fig. 4G-H and Supplementary Fig. S3F-G), demonstrating that targeting the high EDI3 expression in ER − HER2 + cells sensitizes them to lapatinib, even at sub-toxic concentrations. Interestingly, combined inhibition of EDI3 and HER2 appeared to have an additive effect on the viability of SKBR3 cells (Fig. 4E and Supplementary Fig. S3D). In contrast, combined inhibition of HER2 and EDI3 in lapatinib-resistant HCC1954 cells was not significantly different from silencing EDI3 alone under most conditions tested (Fig. 4F and Supplementary Fig. S3E), suggesting that EDI3 may be an alternative target for HER2-targeted therapy resistant tumours.

To further investigate EDI3 as a potential target, we treated cells with the monoclonal antibody trastuzumab, which binds to the extracellular domain of HER2 and inhibits its intracellular signalling and extracellular shedding [32], together with EDI3 silencing. While there was no major effect of trastuzumab on cell viability in any of the four cell lines tested (Supplementary Fig. S3H), SKBR3 and BT474 cells exhibited an approximately 20% viability loss with the concentrations used, which is similar to previous reports categorizing them as sensitive [18]. Conversely, HCC1954 and EFM192A cells are considered nonresponsive and less responsive, respectively [18]. Similar to the results obtained with lapatinib, combined inhibition of EDI3 and HER2 with siRNA and trastuzumab, respectively, had a greater effect on viability than trastuzumab alone in ER-HER2 + cells (SKBR3 and HCC1954; Supplementary Fig. S3I-J). Conversely, in ER + HER2 + cells, this effect was either absent (BT474; Supplementary Fig. S3K), or, although significant, not to the same extent (EFM192A; Supplementary Fig. S3L) as observed in ER-HER2 + cells. In addition, while the combined inhibition of EDI3 and HER2 resulted in a dramatic loss in viability in the trastuzumab-resistant HCC1954 cells, this effect appears to be primarily due to the decrease in EDI3, further indicating that EDI3 may be a relevant target in ER-HER2 + tumours that are resistant to HER2-targeted therapy.

### Inhibiting EDI3 with dipyridamole alone or in combination with HER2-targeted therapy decreases tumour growth in mice

Since silencing EDI3 alone, or in combination with HER2 inhibition, led to a significant decrease in viability, we examined whether this would also hold true for tumour growth in vivo. Currently, there are no specific EDI3 inhibitors, but our previous work showed that the general phosphodiesterase (PDE) inhibitor dipyridamole inhibits EDI3 [11]. PDE inhibitors have been suggested as therapeutic agents for various cancer types [33, 34], including dipyridamole, which has been used in preclinical and clinical studies [35, 36]. We first showed that dipyridamole does indeed inhibit EDI3 activity in HER2 + cells in vitro (Supplementary Fig. S4A-D), confirming our earlier findings [11]. We then examined the effect of dipyridamole on viability. While dipyridamole elicited a dose-dependent decrease in viability in all cell lines, the EC50 values were lower in SKBR3 and HCC1954 compared to BT474 and EFM192A cells (Supplementary Fig. S4E), indicating once more that ER − HER2 + cells are more sensitive to EDI3 inhibition, which is comparable to our findings with specific EDI3 siRNAs.

**Fig. 5:**
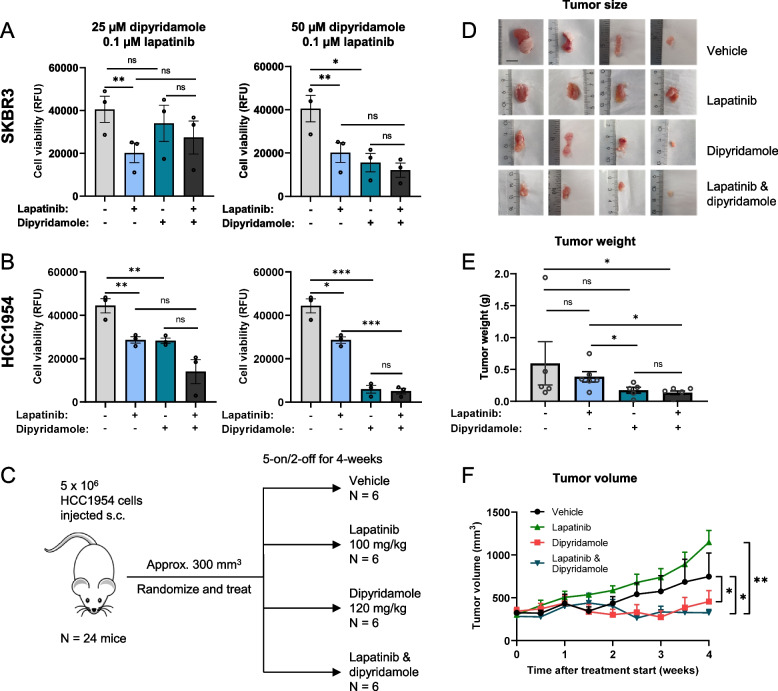
Inhibiting EDI3 with dipyridamole, alone or in combination with HER2-targeting therapy in ER-HER2 + tumours in mice. Viability (RFU) after treating **A,** SKBR3 and **B,** HCC1954 cells with 25 µM (left panel) or 50 µM (right panel) dipyridamole with and without 0.1 µM lapatinib. **C,** Overview of in vivo experimental plan in CD1 nude mice. Mice with HCC1954 subcutaneous tumours were treated with dipyridamole, lapatinib or the combined treatment on a 5-days on/2-days off schedule for 4 weeks and the effect of treatment on tumour **D,** size, **E,** weight, and **F,** volume relative to vehicle control were examined. All in vitro data are mean ± SE of three independent experiments. Measurements (E–F) represent the average tumour weight and volume of four to six mice per condition. (*, *P* < 0.05; **, *P *< 0.01; ***, *P* < 0.001, ns, not significant). RFU, relative fluorescence units

Next, we investigated the influence of combined targeting of EDI3 and HER2 on the viability of SKBR3 and HCC1954 cells. Treating both cell lines with dipyridamole alone at a given concentration led to a greater loss in viability in the lapatinib-resistant HCC1954, compared to the sensitive SKBR3 cells (Fig. 5A-B), with an almost complete loss in viability at 50 µM in the former cell line. The addition of lapatinib had no further impact on viability compared to the effect of each inhibitor alone. As previously observed, lapatinib alone was more effective at decreasing viability in the sensitive SKBR3 compared with the less responsive HCC1954 cells; however, the addition of dipyridamole did not significantly alter the viability of SKBR3 cells (Fig. 5A-B).

Finally, we investigated whether inhibiting EDI3 with dipyridamole would decrease tumour growth of the lapatinib-resistant HCC1954 cells in immunodeficient mice. In addition, we combined dipyridamole with lapatinib to determine whether there would be any advantage upon administering both drugs together in vivo, which was not observed in vitro. Treatment began approximately three weeks after the implantation of cells, and mice were dosed daily for four weeks (5 days on/2 days off) (Fig. 5C) with 100 mg/kg lapatinib [37] and 120 mg/kg dipyridamole. The dose of dipyridamole was selected based on concentrations used in previous work [38, 39] and our own pharmacokinetic study showing that we were able to reach therapeutic concentrations in plasma (0.8–1.2 µg/ml) [40] with a single oral dose of 120 mg/kg (Supplementary Fig. S4F). Treatment of tumour-bearing mice with each compound alone or in combination did not cause weight loss (Supplementary Fig. S4G) or obvious signs of toxicity. Lapatinib alone had no effect on tumour size, weight, or volume (Fig. 5D-F, and Supplementary Fig. S4H), despite evidence of lapatinib in the blood and tumours at concentrations shown to inhibit HER2 activity and decrease viability in vitro (Supplementary Fig. S4I). This is in line with some previous studies showing no effect of lapatinib on HCC1954 tumour growth [41], although others have reported less growth [37]. In contrast, dipyridamole treatment alone significantly decreased tumour size, volume, and weight (although the latter was not significant) of HCC1954 xenografts (Fig. 5D-F and Supplementary Fig. S4H), which was not improved with lapatinib co-treatment. The concentration of dipyridamole in the blood and tumours (Supplementary Fig. S4I) was lower than that which inhibited EDI3 activity and viability in vitro but was nevertheless within the therapeutic range, as previously reported [40]. Therefore, our results indicate that dipyridamole alone may sufficiently reduce ER − HER2 + tumour growth in vivo and support EDI3 as a potential therapeutic target in tumours of this molecular subtype that are resistant to HER2-targeting therapy.

### *Inducibly silencing EDI3 *in vivo* also decreases tumour growth in mice*

**Fig. 6:**
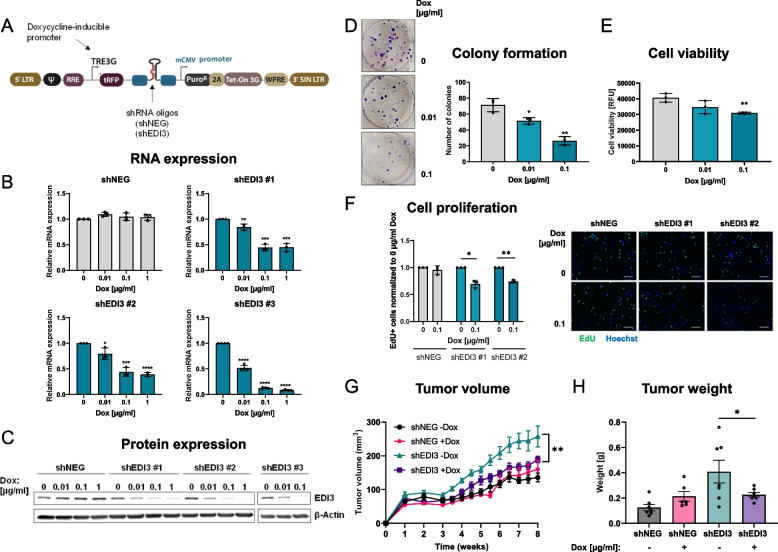
In vitro characterization of inducible EDI3 silencing and its effect on ER-HER2 + tumours in mice. **A,** Overview of the elements of the inducible lentiviral shRNA vector (SMARTvector™, Dharmacon) containing shRNA oligos under the control of a doxycycline inducible promotor. **B,** mRNA expression of EDI3 and **C,** representative Western blot showing EDI3 protein expression in HCC1954 shNEG and HCC1954 shEDI3 (oligos shEDI3 #1, #2, #3) cells treated with 0.01, 0.1 or 1 µg/ml doxycycline for 72 h. **D,** Representative images (left) and corresponding quantification (right) of colonies formed by HCC1954 shEDI3 cells treated with 0.1 or 1 µg/ml doxycycline. **E,** Viability (RFU) after treating HCC1954 shEDI3 cells with 0.1 or 1 µg/ml doxycycline. **F,** Number of EdU positive cells normalized to the control condition (0 μg/ml doxycycline) and representative images (right) after treatment of HCC1954 shNEG and HCC1954 shEDI3 (oligos shEDI3 #1, #2) cells with 10 nM EdU for 2 h followed by labelling with Alexa FluorTM 488 dye (green). Nuclei were stained with Hoechst 33342 (blue), scale bars represent 200 μM. CD1 nude mice were injected subcutaneously with HCC1954 shNEG or HCC1954 shEDI3 cells with (+ Dox) and without (-Dox) doxycycline treatment (0.1 µg/ml) and the effect of silencing EDI3 on tumour **G,** volume and **H,** weight were examined. All in vitro data are mean ± SD of three independent experiments. Measurements (**G**-**H**) represent the average tumour volume and weight of six to seven mice per condition. (*, *P* < 0.05; **, *P* < 0.01; ***, *P* < 0.001, ****, *P* < 0.0001). EdU, 5-Ethynyl-2′-deoxyuridine; RFU, relative fluorescence units

Since dipyridamole does not specifically inhibit EDI3, we validated the observed reduction in tumour volume using a second approach in which EDI3 is exclusively targeted. We created an HCC1954 cell line in which we could inducibly silence EDI3 by the addition of doxycycline (Fig. 6A). Initial in vitro characterization of these cells revealed a doxycycline-dependent decrease in expression (Fig. 6B-C), as well as in colony formation (Fig. 6D) and viability (Fig. 6E), which were confirmed with additional shRNA oligos (Supplemental Figure S5). We could also further confirm that the decrease in viability was due to reduced proliferation (Fig. 6F). To then investigate the effect of specifically silencing EDI3 on tumour growth in vivo, the HCC1954 shNEG and HCC1954 shEDI3 cells were used to create subcutaneous tumours in CD1 nude mice. Similar to the results obtained with dipyridamole, we observed a significant decrease in tumour volume in response to EDI3 silencing upon administration of doxycycline (Fig. 6G). In addition, specifically silencing EDI3 led to a significant decrease in tumour weight (Fig. 6H).

## Discussion

In recent years, there has been tremendous progress in the treatment of HER2 + breast cancer, which looks beyond targeting HER2 alone. Rather, HER2-targeting drugs are either combined with, or more recently conjugated to existing chemotherapies directed against other proteins and pathways deregulated in HER2 breast cancer [42]. In the present work, we show that the glycerophosphodiesterase EDI3 is highly expressed in ER − HER2 + breast cancers, and that downregulating EDI3 in ER − HER2 + cells decreases viability more efficiently compared to those that are ER + HER2 + . Furthermore, we demonstrate that inhibiting or silencing EDI3 slows the growth of xenografts made with HCC1954 cells, which are reported to be less responsive or resistant to HER2-targeting therapies [18].

The present work demonstrates that EDI3 expression is regulated by HER2 signalling, and that inhibiting HER2 and key proteins downstream of HER2, namely GSK3β and mTOR, reduce EDI3 expression. Proteins activated as a result of HER2 signalling, including GSK3β and mTOR, are currently being evaluated in breast cancer with mixed outcomes. For example, GSK3β regulates multiple cellular processes and has both tumour-suppressing and tumour-activating functions [43], making its role in tumour development unclear. However, it has been explored as a potential therapeutic target [43] with limited success [43, 44]. Inhibiting GSK3β in the current work not only decreased EDI3 expression, but also increased the levels of β-catenin. Interestingly, β-catenin activation has been shown to be associated with poor outcome in breast cancer [45], and moreover has been reported to induce cancer stem cell generation [46], suggesting that it may not be a suitable target for therapeutic intervention. Conversely, mTORC1 inhibitors are in clinical trials for various cancer types [47, 48]; everolimus was approved for hormone receptor-positive breast cancer [49], and more recently used for HER2 + tumours [50, 51]. Interestingly, everolimus together with trastuzumab improved survival in ER − HER2 + breast cancer [51], supporting the approach of inhibiting deregulated proteins downstream of HER2.

The exact mechanism by which mTOR and GSK3β regulates EDI3 is currently unknown. Both signalling pathways appear to be differently regulated by PI3K/Akt signalling; however, there is evidence of cross talk between them. For example, overexpression of GSK3β was shown to activate mTORC1 in MCF7 cells [52]; whereas, the mTORC1 substrate, p70S6K1 phosphorylates GSK3β at Ser9 in the absence of TSC1 or 2 resulting in the inactivation of GSK3β [53]. Therefore, to begin elucidating the transcriptional mechanism controlling EDI3 expression, we used small molecule inhibitors against transcription factors predicted to bind the EDI3 promoter region that are downstream of GSK3β and mTORC1. Interestingly, inhibiting HIF1α, CREB and STAT3 increased EDI3 expression, suggesting a negative regulation of EDI3 by these three transcription factors. In previous studies, GSK3β was shown to phosphorylate and inactivate both CREB and HIF1α [24, 25]. In addition, phosphorylation of KLF5 by GSK3β targets KLF5 for degradation [26]. As a result, inhibiting GSK3β would reverse this inactivation and degradation, leaving CREB, HIF1α and KLF5 available to bind the promoter of their target genes. Similarly, inhibition of GSK3β was shown to increase STAT3 and NFκB expression [27], which would then repress EDI3, based on our results that inhibiting STAT3 and NFκB increases EDI3 expression. However, other studies have reported that STAT3 is positively regulated by mTOR and GSK3β [54, 55], suggesting that inhibiting mTOR or GSK3β would decrease STAT3 and consequently increase EDI3 expression. This contradicts the findings in the present work demonstrating reduced EDI3 expression upon inhibition of mTOR or GSK3β. Therefore, while the current results provide some insight into the regulatory factors controlling EDI3 expression, further investigations and experimental approaches are needed to conclusively elucidate how EDI3 is regulated in HER2 positive breast cancer, and the consequence of having high EDI3 expression in this specific subtype.

To begin addressing the significance of elevated EDI3 levels downstream of the HER2 signalling pathway, we hypothesized that its inhibition may support the cytotoxic effect of HER2 antagonizing compounds without the side effects obtained by inhibiting central signalling hubs. If so, this effect should be strongest in the ER − HER2 + molecular subtype with the highest EDI3 expression. Indeed, silencing or inhibiting EDI3 in ER − HER2 + cell lines significantly decreased cell viability. The effect was stronger in HCC1954 cells that contain the PIK3CA H1047R activating mutation shown to be associated with decreased pathological complete response in HER2 breast cancer patients treated with combined trastuzumab and lapatinib [56] and increased resistance to HER2-targeting drugs [18]. Thus, while EDI3 expression seems to correlate with HER2 expression, its effect on cell viability might depend on PIK3CA mutation status. Our observation that inhibiting EDI3 decreased the viability of HCC1954 cells led us to hypothesize that EDI3 is a potential therapeutic target in ER − HER2 + breast cancers resistant to standard therapy. Resistance to commonly used anti-HER2 drugs, such as the monoclonal antibody trastuzumab and the small molecule inhibitor lapatinib, can occur by a multitude of different mechanisms. For example, some resistance mechanisms are relevant for a specific drug, such as the expression of truncated HER2, which lacks the extracellular trastuzumab-binding domain [57]. In contrast, others are common for several drugs and affect factors downstream of the receptor, including the activation of PI3K pathway, e.g. via oncogenic activating *PIK3CA* mutations [58, 59], and loss of PTEN [19, 59]. These altogether make targeting EDI3, which is further downstream of this signalling pathway, an attractive option. Furthermore, acquired resistance owing to the upregulation of other tyrosine kinase receptors, such as EGFR, HER3 and Met [60–62] is often due to the activation of the same downstream pathways as for the HER family receptors, primarily PI3K/Akt/mTOR, which further supports EDI3 as a potentially interesting therapeutic target in this setting. The effect of EDI3 inhibition on resistance mechanisms related with the immune response, such as the escape from trastuzumab-induced antibody-dependent cell-mediated cytotoxicity, or trastuzumab-mediated upregulation of programmed cell death protein 1 (PD-1) [63] and programmed death-ligand 1 (PD-L1) [64] is more difficult to anticipate. Not much is known about the role of EDI3 in different immune cell populations, but databases, such as the Human Protein Atlas web-based resource (https://www.proteinatlas.org/) indicate that EDI3 expression is elevated in several types of immune cells, in particular macrophages, as well as in T and B/plasma cells, which are known to be associated with better prognosis in breast cancer [65]. Consequently, the effect of EDI3 inhibition on effector immune cell function should be clarified.

Recent publications indicate that in addition to HER2 status, the level of HER2 amplification can also influence the response to HER2-targeted therapy [66–68], demonstrating better complete pathological response to neoadjuvant chemotherapy in HER2 positive patients with a higher HER2/CEP17 ratio. However, in the present study, we did not observe higher ERBB2 expression in the anti-HER2 treatment sensitive cell lines compared to cell lines reported to be resistant. We also did not observe higher expression of ERBB2 in ER − HER2 + compared to ER + HER2 + cell lines and tumours, which speaks against HER2 amplification level and/or expression driving the observed difference in EDI3 expression between these two subtypes. Thus, it remains unknown why EDI3 levels are lower in the ER + subtype. If the lower EDI3 expression is due to a negative regulation via ER signalling should be explored in subsequent studies by targeting the ER in ER+HER2 + cells.

The observation that inhibiting EDI3 with dipyridamole decreased the viability of HCC1954 cells prompted us to explore the effect of EDI3 inhibition on tumour growth in vivo. Dipyridamole is used as prophylaxis against thromboembolic events and stroke because of its ability to prevent platelet aggregation and improve vasodilation [69]. It functions as a general phosphodiesterase inhibitor that reduces the breakdown of second messengers, cAMP/cGMP to AMP/GMP [70]. Interestingly, several in vitro and in vivo studies have investigated the efficacy of dipyridamole in different cancer types, either alone [71, 72] or in combination with other drugs [35, 36, 38, 39]. However, the mechanism by which dipyridamole antagonizes tumour growth is unknown. Despite the decrease in tumour growth observed in the present study, dipyridamole is not specific to EDI3. In addition to phosphodiesterases [73], an in-silico study predicted that other proteins may be inhibited by dipyridamole, including pyruvate kinase, peroxisome proliferator-activated receptor gamma (PPARγ), and phosphatidylinositol 4,5-bisphosphate 3-kinase [74]. These candidates were not further investigated in vitro, and while it cannot be excluded that the effect of dipyridamole on tumour growth may partially be due to the inhibition of phosphatidylinositol 4,5-bisphosphate 3-kinase, a separate study [71] showed that dipyridamole had no influence on Akt phosphorylation. To validate that EDI3 is a relevant target in HER2-targeting therapy resistant breast cancer, in a second approach, we silenced EDI3 in the HCC1954 cell line and confirmed decreased tumour growth, as well as tumour weight. While we demonstrate that dipyridamole alone can decrease tumour growth with no obvious side effects, and that the effect seen is specific to EDI3, the identification of a specific EDI3 inhibitor will be a critical next step towards understanding the relevance of EDI3 as a therapeutic target in HER2-targeted therapy resistant breast cancer.

Despite the promising results that EDI3 may be therapeutically relevant in ER-HER2 + breast cancer, there are limitations to the current results. For instance, only xenografts derived from cancer cell lines were used, which are not the most relevant models to predict the clinical benefit of a drug candidate. Key experiments in the future should be repeated using patient-derived xenografts of HER2 + breast cancers resistant to anti-HER2 therapies, representing different resistance mechanisms.

In summary, we show that EDI3 is highly expressed in ER − HER2 + breast cancer, and when inhibited, leads to a significant reduction in viability and tumour growth, particularly in cells resistant to traditional HER2-targeting therapies.

## Supplementary Information

**Additional file 1: Supplementary Figure S1.** EDI3 is highly expressed in ER-HER2+ cells and is regulated by HER2. **A (left panel)**, HER2 mRNA expression measured using qRT-PCR. Quantification of **A (right panel)**, HER2 and **B**, ER protein expression from immunoblotting as represented in Figure 1D. **C**, Immunofluorescence staining, and **D**, metabolite levels by NMR in a panel of breast cancer cell lines representing the different breast cancer subtypes. **E**, HER2 and EDI3 mRNA, and **F**, protein expression in HCC1954 cells upon HER2 silencing. **G**, EDI3 and HER2 mRNA expression and **H**, EDI3, pHER2, and HER2 protein expression in SKBR3 cells after silencing EDI3 with siRNA. **I**, EDI3 and HER2 mRNA expression and **J**, EDI3, pHER2, and HER2 protein expression in HCC1954 cells after silencing EDI3 with siRNA. **K**, Representative Western blots showing EDI3, pHER2 and HER2 protein expression in HCC1954 cells treated with 0.1 µM or 1.0 µM lapatinib for 24, 48, 72 and 96h, with corresponding quantification of protein levels. Data are mean ± SD or mean ± SE for HER2 protein expression in panel A of at least three independent experiments (*, *P*<0.05; **, *P* < 0.01; ***, *P* < 0.001; ****, *P* < 0.0001; ns, not significant).

**Additional file 2: Supplementary Figure S2.** GSK3β and mTOR are key proteins in HER2-mediated upregulation of EDI3. **A**, Quantification of HER2, EDI3 and p-Akt protein expression after inhibiting PI3K with 5 and 10 µM LY294002 for 3 and 7 days in MCF7-NeuT cells. **B**, Representative Western blot and HER2, EDI3 and p-PKCα/βII protein expression quantification after inhibiting PLCγ with 1 and 3 µM U73122 for 3 and 7 days in MCF7-NeuT cells. **C**, Representative Western blot and quantification of HER2, EDI3 and p-ERK1/2 protein expression after inhibiting MEK with 5 and 25 µM PD98059 for 3 and 7 days in MCF7-NeuT cells. **D**, HER2, EDI3 and p-mTOR protein expression after inhibiting mTORC1 with 1 and 3 µM everolimus for 3 and 7 days in MCF7-NeuT cells. **E**, HER2, EDI3 and p-mTOR protein expression after inhibiting mTORC1 with 1 and 3 µM everolimus for 1, 2 and 3 days in HCC1954 cells. **F**, HER2, EDI3 and p-mTOR protein expression after inhibiting mTORC1 with 1 and 3 µM everolimus for 1, 2 and 3 days in SKBR3 cells. **G**, HER2, EDI3 and β-catenin protein expression after inhibiting GSKβ with 1 and 2.5 µM CHIR-99021 for 3 and 7 days in MCF7/NeuT cells. **H**, HER2, EDI3 and β-catenin protein expression after inhibiting GSKβ with 1 and 2.5 µM CHIR-99021 for 1, 2 and 3 days in HCC1954 cells. **I**, HER2, EDI3 and β-catenin protein expression after inhibiting GSKβ with 1 and 2.5 µM CHIR-99021 for 1, 2 and 3 days in SKBR3 cells. Graphs D-I are quantification of Western blots exemplified in Fig. 3C-H, respectively. **J**, EDI3 mRNA expression after inhibiting c-Myc with 5 and 10 µM 10074-G5, **K**, NFκB with 5 and 10 µM SC75741, or **L**, KLF5 with 5 and 10 µM SR18662 for 1, 2 and 3 days in SKBR3 cells. Data are mean ± SD of at least three independent experiments (*, *P*<0.05; **, *P* < 0.01; ***, *P* < 0.001; ****, *P* < 0.0001; ns, not significant). * in MCF7-NeuT cells represents difference from untreated control; # represents difference to cells treated with dox alone.

**Additional file 3: Supplementary Figure S3.** Effect of targeting EDI3 on viability, alone or in combination with lapatinib, in ER-HER2+ and ER+HER2+ breast cancer cell lines. **A-B,** EDI3 mRNA (left panels) and protein expression (right panels) after silencing EDI3 using siRNA in **A, **BT474 and **B, **EFM192A cells.** C, **Effect of inhibiting HER2 using increasing concentrations of lapatinib on viability in SKBR3, HCC1954, BT474 and EFM192A cells. **D-G **Influence of silencing EDI3 with siRNA (oligo #2) and inhibiting HER2 with lapatinib (0.01 µM, 0.1 µM and 1 µM), as well as the combined inhibition of both EDI3 and HER2 on viability in **D, **SKBR3, **E,** HCC1954, **F, **BT474 and **G,** EFM192A cells. **H, **Effect of inhibiting HER2 using different concentrations of trastuzumab on viability in SKBR3, HCC1954, BT474 and EFM192A cells. **I-L** Influence of inhibiting EDI3 with siRNA (oligos #1 and #2) and HER2 with trastuzumab (1 µg/ml and 10 µg/ml), as well as the combined inhibition of both EDI3 and HER2 on viability in **I, **SKBR3, **J, **HCC1954, **K, **BT474 and **L,** EFM192A. Data are mean ± SD (**A**-**C** and **H**) or mean ± SE (**D**-**G** and **I**-**L**) of at least three independent experiments (*, *P* <0.05; **, *P* < 0.01; ***, *P* < 0.001; ****P < 0.0001; ns, not significant).

**Additional file 4: Supplementary Figure S4.** Inhibiting EDI3 with dipyridamole, alone or in combination with HER2-targeting therapy, in ER-HER2+ tumors in mice. **A**-**D **EDI3 activity in percent after treatment with dipyridamole at different concentrations in **A,** HCC1954 **B,** SKBR3, **C,** BT474 and **D,** EFM192 cells. **E, **Cell viability in percent of vehicle control after treatment with different concentrations of dipyridamole in SKBR3, HCC1954, BT474 and EFM192A cells (upper panel) and EC50-values with corresponding 95% confidence intervals (CI) (lower panel). **F, **Concentration of dipyridamole in plasma after a single oral dose of 120 mg/kg dipyridamole in mice over time. **G, **Mouse weight (grams) after treatment with dipyridamole, lapatinib and the combination for up to 4 weeks. **H, **Images of CD1 nude mice with tumors encircled, after 4-week treatment of vehicle, dipyridamole, lapatinib and the combination of both. **I, **Plasma and tumor concentrations (µM) of dipyridamole (upper panel) and lapatinib (lower panel) after treatment with each compound alone or in combination (D+L). Data (**A**-**E**) are mean ± SD of three independent experiments; mean ± SD of three CD1 mice (**F**); mean ± SD 5 to 6 mice (**G**); and representative images of 4 mice per condition **H**. D+L (combined dipyridamole and lapatinib treatment); BQL (below quantification limit); EC50 (half maximal effective concentration).

**Additional file 5: Supplemental Figure S5**. Inducibly silencing EDI3 inhibits colony formation and cell viability.** A, **Representative images (top) and corresponding quantification (bottom) of colony number (left) and size (right) formed by HCC1954 shNEG and HCC1954 shEDI3 (oligos shEDI3 #1, #2, #3) cells treated with 0 or 0.1 µg/ml doxycycline. **B**, Viability (RFU) after treating HCC1954 shNEG and HCC1954 shEDI3 (oligos shEDI3 #1, #2) with 0.1 or 1 µg/ml doxycycline. All in vitro data are mean ± SD of three independent experiments. (*, *P* < 0.05; **, *P* < 0.01; ***, *P* < 0.001). RFU, relative fluorescence units.

**Additional file 6: Supplementary Table S1.** EDI3 (GPCPD1) expression in human breast cancer tissue was evaluated in publicly available Affymetrix HG U133 Plus 2.0 gene expression microarray dataset that were downloaded from the Gene Expression Omnibus webportal (GEO) together with available clinicopathological data.

**Additional file 7: Supplementary Table S2.** List of used reagents including **A**, QuantiTect primer assays, **B**, siRNA and shRNA oligos, and **C**, antibodies.

**Additional file 8: Supplementary Table S3.** Association of EDI3 expression for the three probesets on the Affymetrix HG U133 Plus 2.0 array and available clinicopathological parameters shown for **A**, the six datasets combined, as well as separately for **B**, GSE16446, **C**, GSE19615, **D**, GSE28844, **E**, GSE32646, **F**, GSE6532, and **G**, GSE9195. P value from the Mann Whitney or Kruskal-Wallis U test.

## Data Availability

The Affymetrix gene expression microarray datasets analysed in the current study (GSE16446, GSE19615, GSE28844, GSE32646, GSE6532, and GSE6532) were downloaded from the Gene Expression Omnibus (GEO) repository, [https://www.ncbi.nlm.nih.gov/geo/]. All relevant data are available from the authors upon request.

## Abbreviations

CHKA: Choline kinase alpha
CREB: CAMP response element-binding protein
EDI3: Endometrial carcinoma differential 3
ERETIC: Electronic reference to access in vivo concentrations
ER: Estrogen receptor
G3P: Glycerol-3-phosphate
GDE: Glycerophosphodiesterase
GEO: Gene Expression Omnibus
GPC: Glycerophosphocholine
GPCPD1: GPC-phosphodiesterase 1
GSK3β: Glycogen synthase kinase 3 beta
HER2: Epidermal growth factor receptor 2
HIF1α: Hypoxia-induced factor 1α
IHC: Immunohistochemistry
KLF5: Krüppel-like factor 5
LC–MS: Liquid chromatography-mass spectrometry
mTOR: Mechanistic target of rapamycin
NFκB: Nuclear factor κB
NMR: Nuclear magnetic resonance
PCho: Phosphocholine
PDE: Phosphodiesterase
PI3K: Phosphatidylinositol 3-kinase
PKCα/βII: Protein kinase C alpha/beta
PLCγ: Phospholipase C gamma
PtdCho: Phosphatidylcholine
RNAi: RNA interference
siRNA: Silencing RNA
STAT3: Signal transducer and activator of transcription 3
tCho: Total choline
TMA: Tissue microarray

## References

[1] Rexer BN, Arteaga CL (2012). Intrinsic and acquired resistance to HER2-targeted therapies in HER2 gene-amplified breast cancer: mechanisms and clinical implications. Crit Rev Oncog.

[2] Luengo A, Gui DY, Vander Heiden MG (2017). Targeting metabolism for cancer therapy. Cell Chem Biol.

[3] Glunde K, Bhujwalla ZM, Ronen SM (2011). Choline metabolism in malignant transformation. Nat Rev Cancer.

[4] Iorio E, Caramujo MJ, Cecchetti S, Spadaro F, Carpinelli G, Canese R (2016). Key players in choline metabolic reprograming in triple-negative breast cancer. Front Oncol.

[5] Sonkar K, Ayyappan V, Tressler CM, Adelaja O, Cai R, Cheng M (2019). Focus on the glycerophosphocholine pathway in choline phospholipid metabolism of cancer. NMR Biomed.

[6] Aboagye EO, Bhujwalla ZM (1999). Malignant transformation alters membrane choline phospholipid metabolism of human mammary epithelial cells. Cancer Res.

[7] Chen JH, Mehta RS, Baek HM, Nie K, Liu H, Lin MQ (2011). Clinical characteristics and biomarkers of breast cancer associated with choline concentration measured by 1H MRS. NMR Biomed.

[8] Glunde K, Jie C, Bhujwalla ZM (2004). Molecular causes of the aberrant choline phospholipid metabolism in breast cancer. Cancer Res.

[9] Mori N, Wildes F, Takagi T, Glunde K, Bhujwalla ZM (2016). The tumor microenvironment modulates choline and lipid metabolism. Front Oncol.

[10] RamírezdeMolina A, Gutiérrez R, Ramos MA, Silva JM, Silva J, Bonilla F (2002). Increased choline kinase activity in human breast carcinomas: clinical evidence for a potential novel antitumor strategy. Oncogene..

[11] Stewart JD, Marchan R, Lesjak MS, Lambert J, Hergenroeder R, Ellis JK (2012). Choline-releasing glycerophosphodiesterase EDI3 drives tumor cell migration and metastasis. Proc Natl Acad Sci U S A.

[12] Lesjak MS, Marchan R, Stewart JD, Rempel E, Rahnenführer J, Hengstler JG (2014). EDI3 links choline metabolism to integrin expression, cell adhesion and spreading. Cell Adh Migr.

[13] Huang KB, Pan YH, Shu GN, Yao HH, Liu X, Zhou M (2021). Circular RNA circSNX6 promotes sunitinib resistance in renal cell carcinoma through the miR-1184/GPCPD1/ lysophosphatidic acid axis. Cancer Lett.

[14] Marchan R, Büttner B, Lambert J, Edlund K, Glaeser I, Blaszkewicz M (2017). Glycerol-3-phosphate acyltransferase 1 promotes tumor cell migration and poor survival in ovarian carcinoma. Cancer Res.

[15] McCall MN, Bolstad BM, Irizarry RA (2010). Frozen robust multiarray analysis (fRMA). Biostatistics.

[16] Trost TM, Lausch EU, Fees SA, Schmitt S, Enklaar T, Reutzel D (2005). Premature senescence is a primary fail-safe mechanism of ERBB2-driven tumorigenesis in breast carcinoma cells. Cancer Res.

[17] Faustino-Rocha A, Oliveira PA, Pinho-Oliveira J, Teixeira-Guedes C, Soares-Maia R, da Costa RG (2013). Estimation of rat mammary tumor volume using caliper and ultrasonography measurements. Lab Anim (NY).

[18] Jernström S, Hongisto V, Leivonen SK, Due EU, Tadele DS, Edgren H (2017). Drug-screening and genomic analyses of HER2-positive breast cancer cell lines reveal predictors for treatment response. Breast Cancer (Dove Med Press).

[19] Nagata Y, Lan KH, Zhou X, Tan M, Esteva FJ, Sahin AA (2004). PTEN activation contributes to tumor inhibition by trastuzumab, and loss of PTEN predicts trastuzumab resistance in patients. Cancer Cell.

[20] Collins DM, Madden SF, Gaynor N, AlSultan D, Le Gal M, Eustace AJ (2021). Effects of HER family-targeting tyrosine kinase inhibitors on antibody-dependent cell-mediated cytotoxicity in HER2-expressing breast cancer. Clin Cancer Res.

[21] Hurvitz SA, Kalous O, Conklin D, Desai AJ, Dering J, Anderson L (2015). In vitro activity of the mTOR inhibitor everolimus, in a large panel of breast cancer cell lines and analysis for predictors of response. Breast Cancer Res Treat.

[22] Castro-Mondragon JA, Riudavets-Puig R, Rauluseviciute I, Lemma RB, Turchi L, Blanc-Mathieu R (2022). JASPAR 2022: the 9th release of the open-access database of transcription factor binding profiles. Nucleic Acids Res.

[23] Cartharius K, Frech K, Grote K, Klocke B, Haltmeier M, Klingenhoff A (2005). MatInspector and beyond: promoter analysis based on transcription factor binding sites. Bioinformatics.

[24] Flugel D, Gorlach A, Michiels C, Kietzmann T (2007). Glycogen synthase kinase 3 phosphorylates hypoxia-inducible factor 1alpha and mediates its destabilization in a VHL-independent manner. Mol Cell Biol.

[25] Grimes CA, Jope RS (2001). CREB DNA binding activity is inhibited by glycogen synthase kinase-3 beta and facilitated by lithium. J Neurochem.

[26] Bialkowska AB, Liu Y, Nandan MO, Yang VW (2014). A colon cancer-derived mutant of Kruppel-like factor 5 (KLF5) is resistant to degradation by glycogen synthase kinase 3beta (GSK3beta) and the E3 ubiquitin ligase F-box and WD repeat domain-containing 7alpha (FBW7alpha). J Biol Chem.

[27] Bilsland AE, Hoare S, Stevenson K, Plumb J, Gomez-Roman N, Cairney C (2009). Dynamic telomerase gene suppression via network effects of GSK3 inhibition. PLoS ONE.

[28] Steven A, Friedrich M, Jank P, Heimer N, Budczies J, Denkert C (2020). What turns CREB on? And off? And why does it matter?. Cell Mol Life Sci.

[29] Zhang Y, Zhang H, Wang M, Schmid T, Xin Z, Kozhuharova L (2021). Hypoxia in breast cancer-scientific translation to therapeutic and diagnostic clinical applications. Front Oncol.

[30] Sonnenblick A, Agbor-Tarh D, de Azambuja E, Hultsch S, Izquierdo M, Liu M (2021). STAT3 activation in HER2-positive breast cancers: Analysis of data from a large prospective trial. Int J Cancer.

[31] Wang W, Nag SA, Zhang R (2015). Targeting the NFkappaB signaling pathways for breast cancer prevention and therapy. Curr Med Chem.

[32] Hudis CA (2007). Trastuzumab–mechanism of action and use in clinical practice. N Engl J Med.

[33] Huang W, Sundquist J, Sundquist K, Ji J (2020). Phosphodiesterase-5 inhibitors use and risk for mortality and metastases among male patients with colorectal cancer. Nat Commun.

[34] Peng T, Gong J, Jin Y, Zhou Y, Tong R, Wei X (2018). Inhibitors of phosphodiesterase as cancer therapeutics. Eur J Med Chem.

[35] Isacoff WH, Bendetti JK, Barstis JJ, Jazieh AR, Macdonald JS, Philip PA (2007). Phase II trial of infusional fluorouracil, leucovorin, mitomycin, and dipyridamole in locally advanced unresectable pancreatic adenocarcinoma: SWOG S9700. J Clin Oncol.

[36] Mishra RR, Belder N, Ansari SA, Kayhan M, Bal H, Raza U (2018). Reactivation of cAMP Pathway by PDE4D inhibition represents a novel druggable axis for overcoming tamoxifen resistance in er-positive breast cancer. Clin Cancer Res.

[37] Sun B, Mason S, Wilson RC, Hazard SE, Wang Y, Fang R (2020). Inhibition of the transcriptional kinase CDK7 overcomes therapeutic resistance in HER2-positive breast cancers. Oncogene.

[38] Longo J, Mullen PJ, Yu R, van Leeuwen JE, Masoomian M, Woon DTS (2019). An actionable sterol-regulated feedback loop modulates statin sensitivity in prostate cancer. Mol Metab.

[39] Pandyra A, Mullen PJ, Kalkat M, Yu R, Pong JT, Li Z (2014). Immediate utility of two approved agents to target both the metabolic mevalonate pathway and its restorative feedback loop. Cancer Res.

[40] Venkatesh PK, Pattillo CB, Branch B, Hood J, Thoma S, Illum S (2010). Dipyridamole enhances ischaemia-induced arteriogenesis through an endocrine nitrite/nitric oxide-dependent pathway. Cardiovasc Res.

[41] MacNeil IA, Burns DJ, Rich BE, Soltani SM, Kharbush S, Osterhaus NG (2020). New HER2-negative breast cancer subtype responsive to anti-HER2 therapy identified. J Cancer Res Clin Oncol.

[42] Mezni E, Vicier C, Guerin M, Sabatier R, Bertucci F, Gonçalves A (2020). New therapeutics in HER2-positive advanced breast cancer: towards a change in clinical practices? pi. Cancers (Basel)..

[43] Walz A, Ugolkov A, Chandra S, Kozikowski A, Carneiro BA, O'Halloran TV (2017). Molecular pathways: revisiting glycogen synthase kinase-3β as a target for the treatment of cancer. Clin Cancer Res.

[44] Duda P, Akula SM, Abrams SL, Steelman LS, Martelli AM, Cocco L (2020). Targeting GSK3 and associated signaling pathways involved in cancer. Cells..

[45] Li S, Li S, Sun Y, Li L (2014). The expression of beta-catenin in different subtypes of breast cancer and its clinical significance. Tumour Biol.

[46] Liao S, Gan L, Qin W, Liu C, Mei Z (2018). Inhibition of GSK3 and MEK induced cancer stem cell generation via the Wnt and MEK signaling pathways. Oncol Rep.

[47] Chiarini F, Evangelisti C, McCubrey JA, Martelli AM (2015). Current treatment strategies for inhibiting mTOR in cancer. Trends Pharmacol Sci.

[48] Zou Z, Tao T, Li H, Zhu X (2020). mTOR signaling pathway and mTOR inhibitors in cancer: progress and challenges. Cell Biosci.

[49] Baselga J, Campone M, Piccart M, Burris HA, Rugo HS, Sahmoud T (2012). Everolimus in postmenopausal hormone-receptor-positive advanced breast cancer. N Engl J Med.

[50] Hurvitz SA, Andre F, Jiang Z, Shao Z, Mano MS, Neciosup SP (2015). Combination of everolimus with trastuzumab plus paclitaxel as first-line treatment for patients with HER2-positive advanced breast cancer (BOLERO-1): a phase 3, randomised, double-blind, multicentre trial. Lancet Oncol.

[51] Toi M, Shao Z, Hurvitz S, Tseng LM, Zhang Q, Shen K (2017). Efficacy and safety of everolimus in combination with trastuzumab and paclitaxel in Asian patients with HER2+ advanced breast cancer in BOLERO-1. Breast Cancer Res.

[52] Azoulay-Alfaguter I, Elya R, Avrahami L, Katz A, Eldar-Finkelman H (2015). Combined regulation of mTORC1 and lysosomal acidification by GSK-3 suppresses autophagy and contributes to cancer cell growth. Oncogene.

[53] Zhang HH, Lipovsky AI, Dibble CC, Sahin M, Manning BD (2006). S6K1 regulates GSK3 under conditions of mTOR-dependent feedback inhibition of Akt. Mol Cell.

[54] Beurel E, Jope RS (2008). Differential regulation of STAT family members by glycogen synthase kinase-3. J Biol Chem.

[55] Zhou J, Wulfkuhle J, Zhang H, Gu P, Yang Y, Deng J (2007). Activation of the PTEN/mTOR/STAT3 pathway in breast cancer stem-like cells is required for viability and maintenance. Proc Natl Acad Sci U S A.

[56] Loibl S, von Minckwitz G, Schneeweiss A, Paepke S, Lehmann A, Rezai M (2014). PIK3CA mutations are associated with lower rates of pathologic complete response to anti-human epidermal growth factor receptor 2 (her2) therapy in primary HER2-overexpressing breast cancer. J Clin Oncol.

[57] Sperinde J, Jin X, Banerjee J, Penuel E, Saha A, Diedrich G (2010). Quantitation of p95HER2 in paraffin sections by using a p95-specific antibody and correlation with outcome in a cohort of trastuzumab-treated breast cancer patients. Clin Cancer Res.

[58] Berns K, Horlings HM, Hennessy BT, Madiredjo M, Hijmans EM, Beelen K (2007). A functional genetic approach identifies the PI3K pathway as a major determinant of trastuzumab resistance in breast cancer. Cancer Cell.

[59] Eichhorn PJ, Gili M, Scaltriti M, Serra V, Guzman M, Nijkamp W (2008). Phosphatidylinositol 3-kinase hyperactivation results in lapatinib resistance that is reversed by the mTOR/phosphatidylinositol 3-kinase inhibitor NVP-BEZ235. Cancer Res.

[60] Ritter CA, Perez-Torres M, Rinehart C, Guix M, Dugger T, Engelman JA (2007). Human breast cancer cells selected for resistance to trastuzumab in vivo overexpress epidermal growth factor receptor and ErbB ligands and remain dependent on the ErbB receptor network. Clin Cancer Res.

[61] Shattuck DL, Miller JK, Carraway KL, Sweeney C (2008). Met receptor contributes to trastuzumab resistance of Her2-overexpressing breast cancer cells. Cancer Res.

[62] Yang L, Li Y, Shen E, Cao F, Li L, Li X (2017). NRG1-dependent activation of HER3 induces primary resistance to trastuzumab in HER2-overexpressing breast cancer cells. Int J Oncol.

[63] Varadan V, Gilmore H, Miskimen KL, Tuck D, Parsai S, Awadallah A (2016). Immune signatures following single dose trastuzumab predict pathologic response to preoperativetrastuzumab and chemotherapy in HER2-positive early breast cancer. Clin Cancer Res.

[64] Chaganty BKR, Qiu S, Gest A, Lu Y, Ivan C, Calin GA (2018). Trastuzumab upregulates PD-L1 as a potential mechanism of trastuzumab resistance through engagement of immune effector cells and stimulation of IFNgamma secretion. Cancer Lett.

[65] Schmidt M, Bohm D, von Torne C, Steiner E, Puhl A, Pilch H (2008). The humoral immune system has a key prognostic impact in node-negative breast cancer. Cancer Res.

[66] Choi JH, Jeon CW, Kim YO, Jung S (2020). Pathological complete response to neoadjuvant trastuzumab and pertuzumab therapy is related to human epidermal growth factor receptor 2 (HER2) amplification level in HER2-amplified breast cancer. Med (Baltimore).

[67] Greenwell K, Hussain L, Lee D, Bramlage M, Bills G, Mehta A (2020). Complete pathologic response rate to neoadjuvant chemotherapy increases with increasing HER2/CEP17 ratio in HER2 overexpressing breast cancer: analysis of the National Cancer Database (NCDB). Breast Cancer Res Treat.

[68] Singer CF, Tan YY, Fitzal F, Steger GG, Egle D, Reiner A (2017). Pathological complete response to neoadjuvant trastuzumab is dependent on HER2/CEP17 ratio in HER2-amplified early breast cancer. Clin Cancer Res.

[69] Allahham M, Lerman A, Atar D, Birnbaum Y. Why not dipyridamole: a Review of current guidelines and re-evaluation of utility in the modern era. Cardiovasc Drugs Ther. 2022;36(3):525–32. 10.1007/s10557-021-07224-9 PMC8271326 34245446

[70] Harker LA, Kadatz RA (1983). Mechanism of action of dipyridamole. Thromb Res Suppl.

[71] Spano D, Marshall JC, Marino N, De Martino D, Romano A, Scoppettuolo MN (2013). Dipyridamole prevents triple-negative breast-cancer progression. Clin Exp Metastasis.

[72] Wang C, Schwab LP, Fan M, Seagroves TN, Buolamwini JK (2013). Chemoprevention activity of dipyridamole in the MMTV-PyMT transgenic mouse model of breast cancer. Cancer Prev Res (Phila).

[73] Barone I, Giordano C, Bonofiglio D, Andò S, Catalano S (2017). Phosphodiesterase type 5 and cancers: progress and challenges. Oncotarget.

[74] Ge SM, Zhan DL, Zhang SH, Song LQ, Han WW (2016). Reverse screening approach to identify potential anti-cancer targets of dipyridamole. Am J Transl Res.

