# Supplementary material for: Inhibiting the glycerophosphodiesterase EDI3 in ER-HER2+ breast cancer cells resistant to HER2-targeted therapy reduces viability and tumour growth
Source: J Exp Clin Cancer Res. 2023 Jan 20;42:25. doi: 10.1186/s13046-022-02578-w (PMC9854078; doi:10.1186/s13046-022-02578-w)
Supplement: Supplementary file 1 — Additional file 1: Supplementary Figure S1. EDI3 is highly expressed in ER-HER2+ cells and is regulated by HER2. A (left panel), HER2 mRNA expression measured using qRT-PCR. Quantification of A (right panel), HER2 and B, ER protein expression from immunoblotting as represented in Figure 1D. C, Immunofluorescence staining, and D, metabolite levels by NMR in a panel of breast cancer cell lines representing the different breast cancer subtypes. E, HER2 and EDI3 mRNA, and F, protein expression in HCC1954 cells upon HER2 silencing. G, EDI3 and HER2 mRNA expression and H, EDI3, pHER2, and HER2 protein expression in SKBR3 cells after silencing EDI3 with siRNA. I, EDI3 and HER2 mRNA expression and J, EDI3, pHER2, and HER2 protein expression in HCC1954 cells after silencing EDI3 with siRNA. K, Representative Western blots showing EDI3, pHER2 and HER2 protein expression in HCC1954 cells treated with 0.1 µM or 1.0 µM lapatinib for 24, 48, 72 and 96h, with corresponding quantification of protein levels. Data are mean ± SD or mean ± SE for HER2 protein expression in panel A of at least three independent experiments (*, P<0.05; **, P < 0.01; ***, P < 0.001; ****, P < 0.0001; ns, not significant). [file 13046_2022_2578_MOESM1_ESM.pptx]

## Slide 1
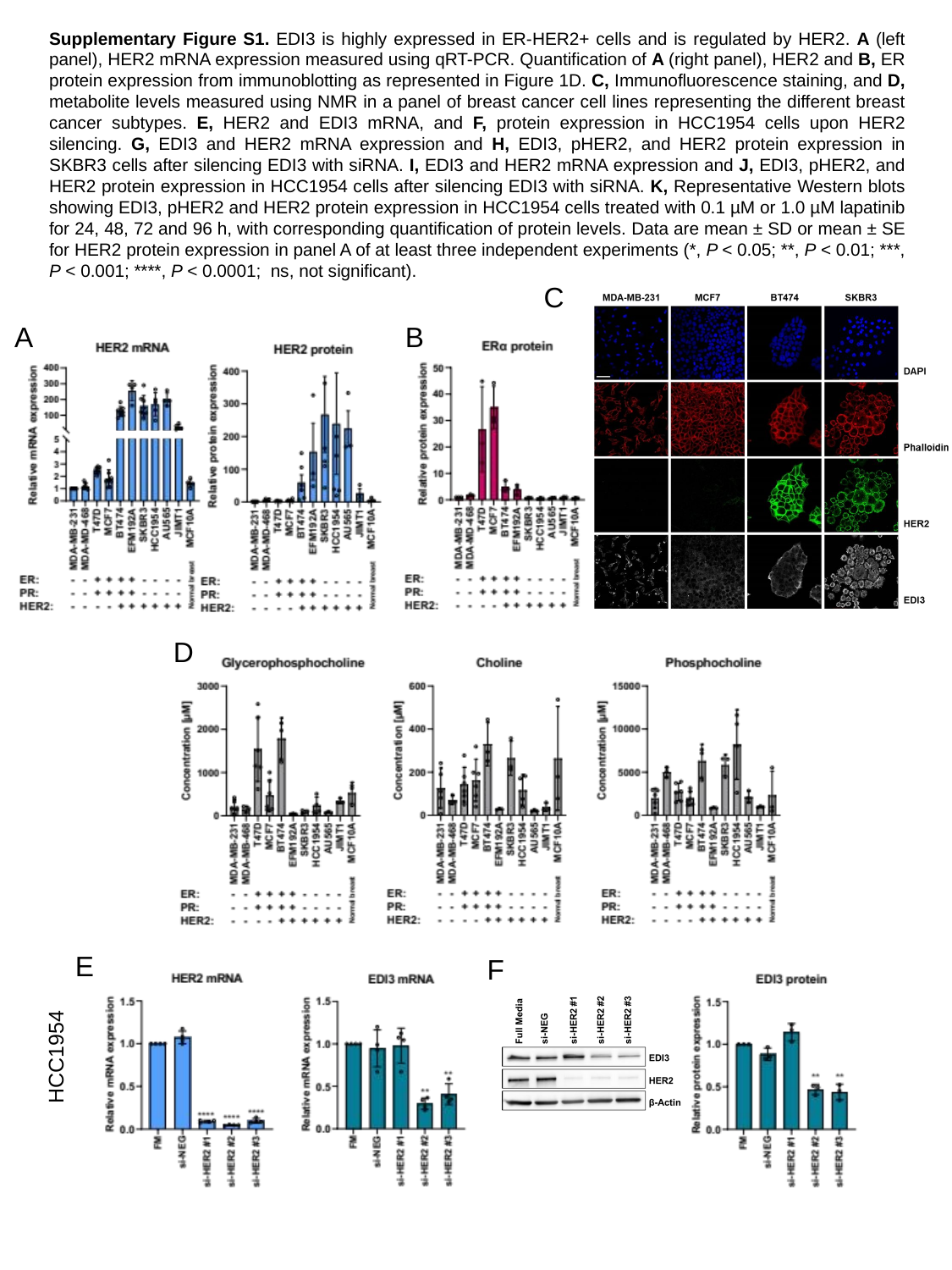

Supplementary Figure S1. EDI3 is highly expressed in ER-HER2+ cells and is regulated by HER2. A (left panel), HER2 mRNA expression measured using qRT-PCR. Quantification of A (right panel), HER2 and B, ER protein expression from immunoblotting as represented in Figure 1D. C, Immunofluorescence staining, and D, metabolite levels measured using NMR in a panel of breast cancer cell lines representing the different breast cancer subtypes. E, HER2 and EDI3 mRNA, and F, protein expression in HCC1954 cells upon HER2 silencing. G, EDI3 and HER2 mRNA expression and H, EDI3, pHER2, and HER2 protein expression in SKBR3 cells after silencing EDI3 with siRNA. I, EDI3 and HER2 mRNA expression and J, EDI3, pHER2, and HER2 protein expression in HCC1954 cells after silencing EDI3 with siRNA. K, Representative Western blots showing EDI3, pHER2 and HER2 protein expression in HCC1954 cells treated with 0.1 µM or 1.0 µM lapatinib for 24, 48, 72 and 96 h, with corresponding quantification of protein levels. Data are mean ± SD or mean ± SE for HER2 protein expression in panel A of at least three independent experiments (*, P < 0.05; **, P < 0.01; ***, P < 0.001; ****, P < 0.0001; ns, not significant).
C
A
B
D
E
F
HCC1954

## Slide 2
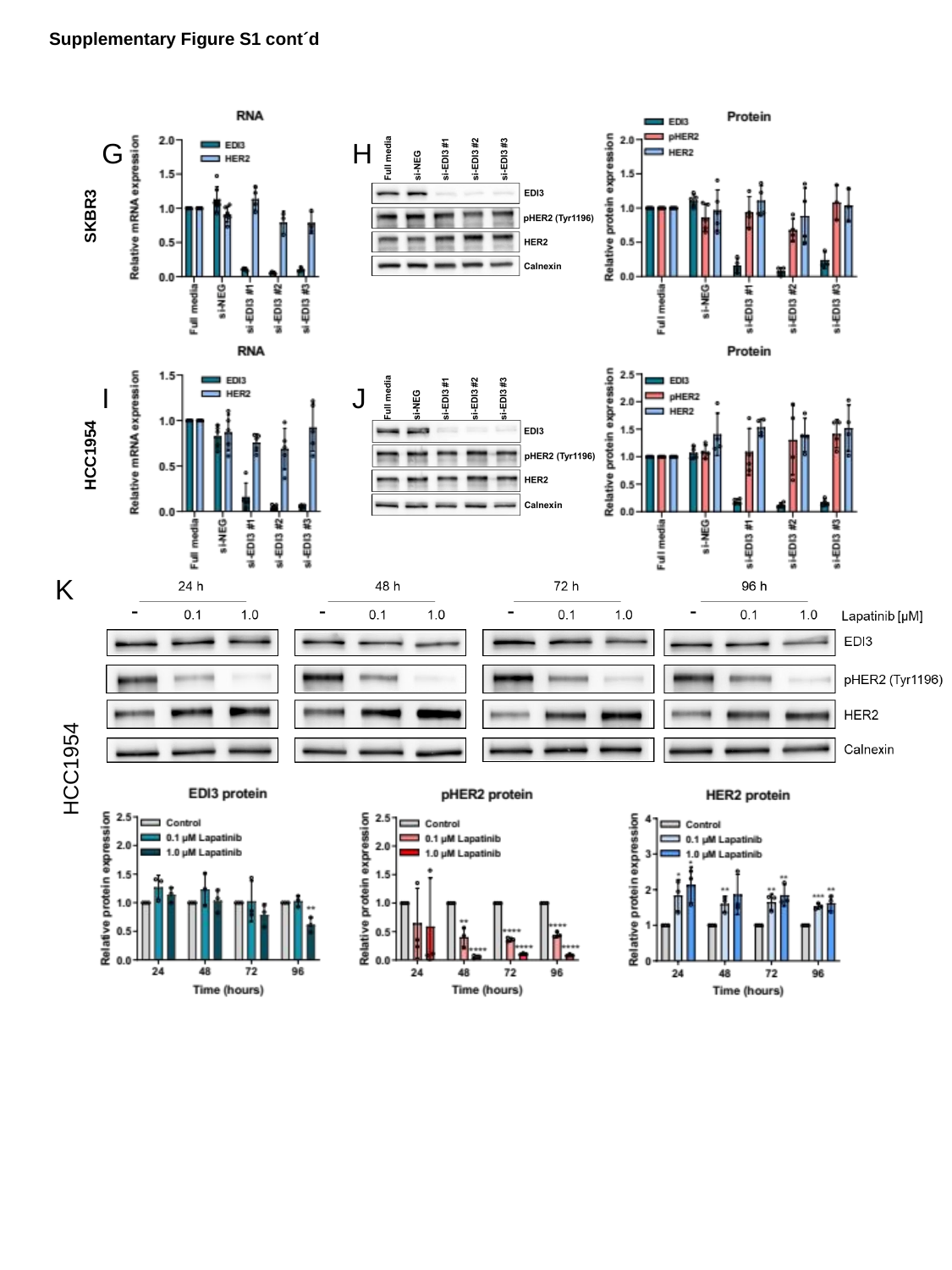

Supplementary Figure S1 cont´d
G
H
SKBR3
I
J
HCC1954
K
HCC1954
