# Supplementary material for: Inhibiting the glycerophosphodiesterase EDI3 in ER-HER2+ breast cancer cells resistant to HER2-targeted therapy reduces viability and tumour growth
Source: J Exp Clin Cancer Res. 2023 Jan 20;42:25. doi: 10.1186/s13046-022-02578-w (PMC9854078; doi:10.1186/s13046-022-02578-w)
Supplement: Supplementary file 2 — Additional file 2: Supplementary Figure S2. GSK3β and mTOR are key proteins in HER2-mediated upregulation of EDI3. A, Quantification of HER2, EDI3 and p-Akt protein expression after inhibiting PI3K with 5 and 10 µM LY294002 for 3 and 7 days in MCF7-NeuT cells. B, Representative Western blot and HER2, EDI3 and p-PKCα/βII protein expression quantification after inhibiting PLCγ with 1 and 3 µM U73122 for 3 and 7 days in MCF7-NeuT cells. C, Representative Western blot and quantification of HER2, EDI3 and p-ERK1/2 protein expression after inhibiting MEK with 5 and 25 µM PD98059 for 3 and 7 days in MCF7-NeuT cells. D, HER2, EDI3 and p-mTOR protein expression after inhibiting mTORC1 with 1 and 3 µM everolimus for 3 and 7 days in MCF7-NeuT cells. E, HER2, EDI3 and p-mTOR protein expression after inhibiting mTORC1 with 1 and 3 µM everolimus for 1, 2 and 3 days in HCC1954 cells. F, HER2, EDI3 and p-mTOR protein expression after inhibiting mTORC1 with 1 and 3 µM everolimus for 1, 2 and 3 days in SKBR3 cells. G, HER2, EDI3 and β-catenin protein expression after inhibiting GSKβ with 1 and 2.5 µM CHIR-99021 for 3 and 7 days in MCF7/NeuT cells. H, HER2, EDI3 and β-catenin protein expression after inhibiting GSKβ with 1 and 2.5 µM CHIR-99021 for 1, 2 and 3 days in HCC1954 cells. I, HER2, EDI3 and β-catenin protein expression after inhibiting GSKβ with 1 and 2.5 µM CHIR-99021 for 1, 2 and 3 days in SKBR3 cells. Graphs D-I are quantification of Western blots exemplified in Fig. 3C-H, respectively. J, EDI3 mRNA expression after inhibiting c-Myc with 5 and 10 µM 10074-G5, K, NFκB with 5 and 10 µM SC75741, or L, KLF5 with 5 and 10 µM SR18662 for 1, 2 and 3 days in SKBR3 cells. Data are mean ± SD of at least three independent experiments (*, P<0.05; **, P < 0.01; ***, P < 0.001; ****, P < 0.0001; ns, not significant). * in MCF7-NeuT cells represents difference from untreated control; # represents difference to cells treated with dox alone. [file 13046_2022_2578_MOESM2_ESM.pptx]

## Slide 1
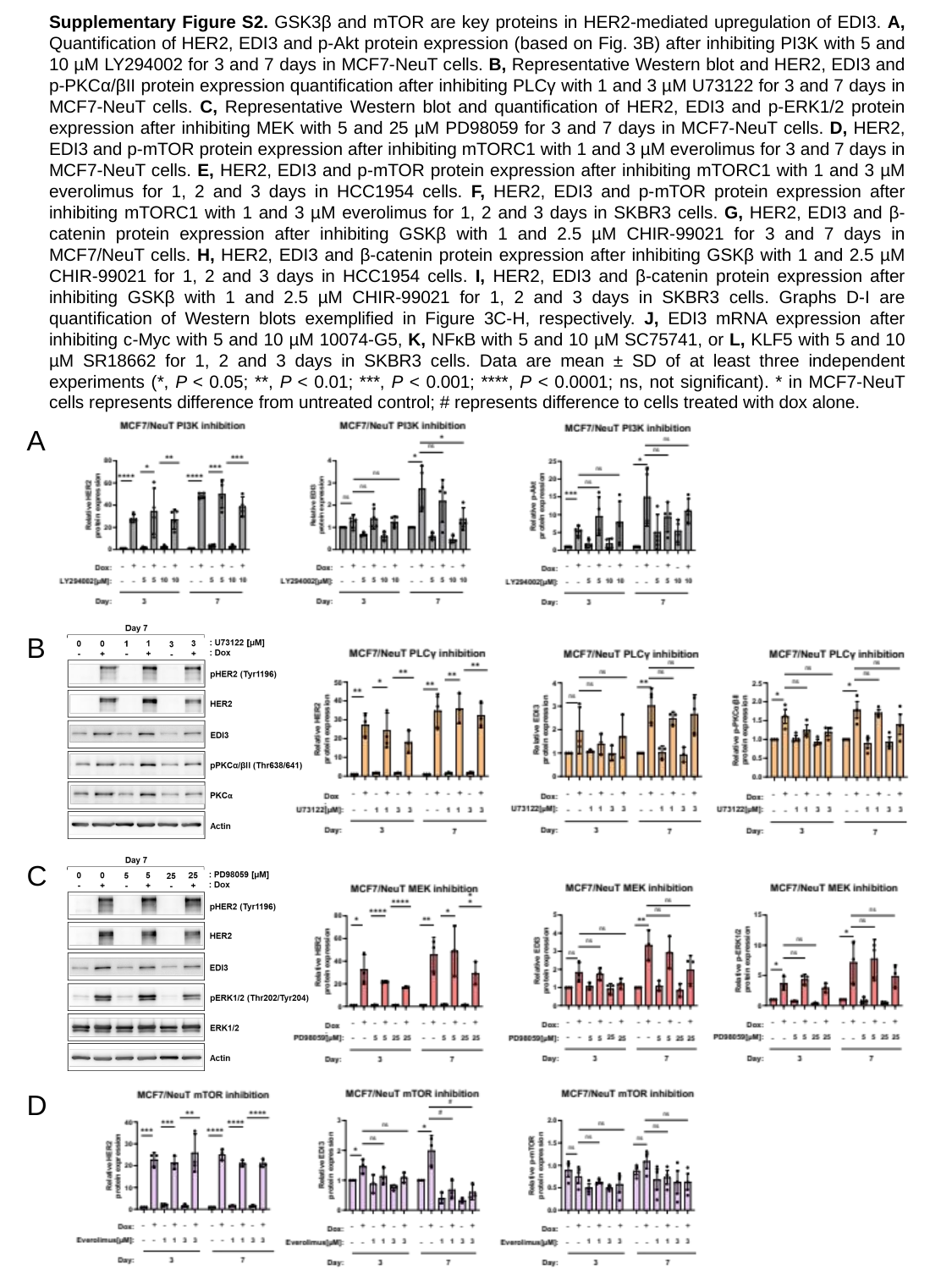

Supplementary Figure S2. GSK3β and mTOR are key proteins in HER2-mediated upregulation of EDI3. A, Quantification of HER2, EDI3 and p-Akt protein expression (based on Fig. 3B) after inhibiting PI3K with 5 and 10 µM LY294002 for 3 and 7 days in MCF7-NeuT cells. B, Representative Western blot and HER2, EDI3 and p-PKCα/βII protein expression quantification after inhibiting PLCγ with 1 and 3 µM U73122 for 3 and 7 days in MCF7-NeuT cells. C, Representative Western blot and quantification of HER2, EDI3 and p-ERK1/2 protein expression after inhibiting MEK with 5 and 25 µM PD98059 for 3 and 7 days in MCF7-NeuT cells. D, HER2, EDI3 and p-mTOR protein expression after inhibiting mTORC1 with 1 and 3 µM everolimus for 3 and 7 days in MCF7-NeuT cells. E, HER2, EDI3 and p-mTOR protein expression after inhibiting mTORC1 with 1 and 3 µM everolimus for 1, 2 and 3 days in HCC1954 cells. F, HER2, EDI3 and p-mTOR protein expression after inhibiting mTORC1 with 1 and 3 µM everolimus for 1, 2 and 3 days in SKBR3 cells. G, HER2, EDI3 and β-catenin protein expression after inhibiting GSKβ with 1 and 2.5 µM CHIR-99021 for 3 and 7 days in MCF7/NeuT cells. H, HER2, EDI3 and β-catenin protein expression after inhibiting GSKβ with 1 and 2.5 µM CHIR-99021 for 1, 2 and 3 days in HCC1954 cells. I, HER2, EDI3 and β-catenin protein expression after inhibiting GSKβ with 1 and 2.5 µM CHIR-99021 for 1, 2 and 3 days in SKBR3 cells. Graphs D-I are quantification of Western blots exemplified in Figure 3C-H, respectively. J, EDI3 mRNA expression after inhibiting c-Myc with 5 and 10 µM 10074-G5, K, NFκB with 5 and 10 µM SC75741, or L, KLF5 with 5 and 10 µM SR18662 for 1, 2 and 3 days in SKBR3 cells. Data are mean ± SD of at least three independent experiments (*, P < 0.05; **, P < 0.01; ***, P < 0.001; ****, P < 0.0001; ns, not significant). * in MCF7-NeuT cells represents difference from untreated control; # represents difference to cells treated with dox alone.
A
B
C
D

## Slide 2
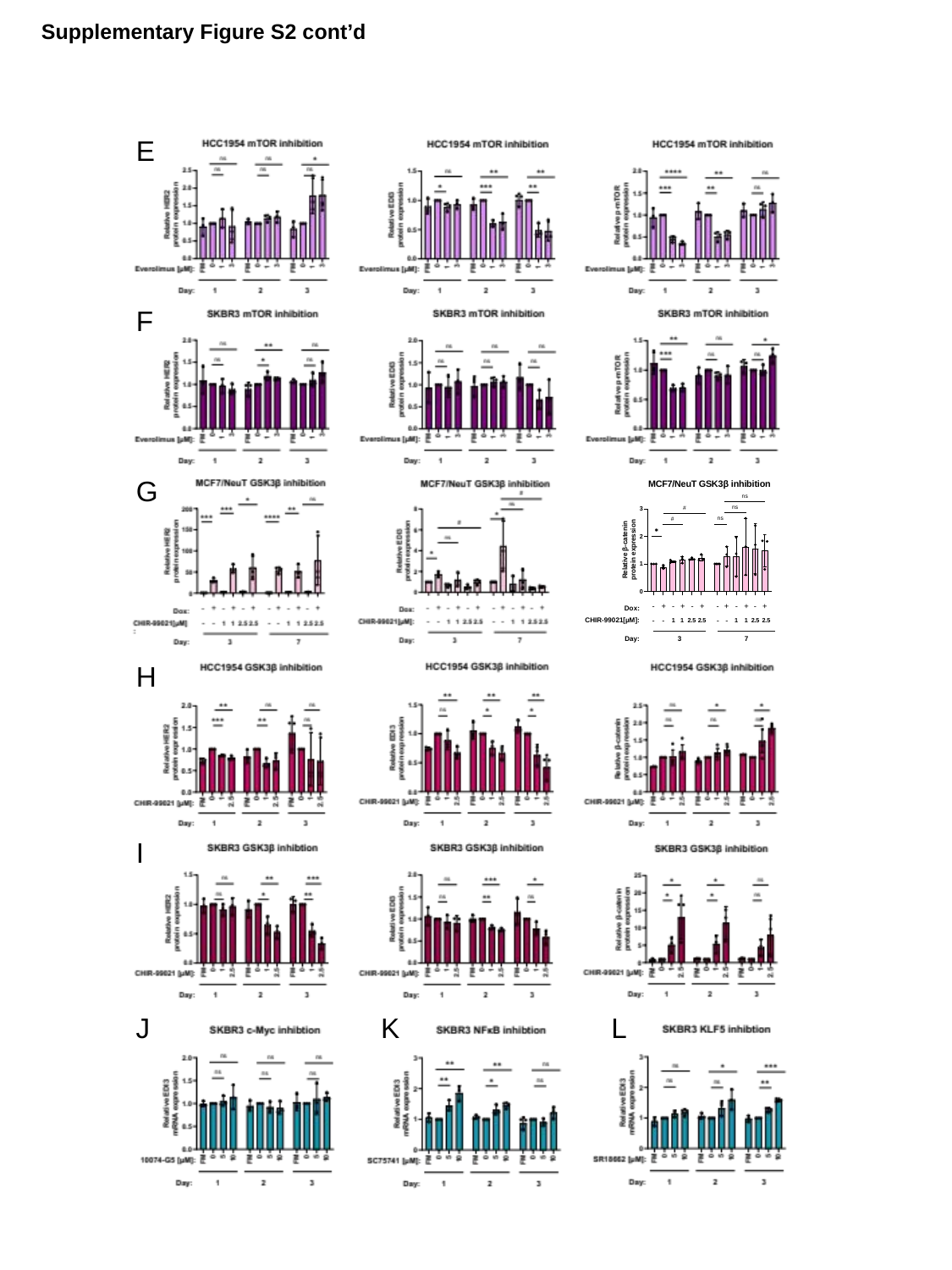

Supplementary Figure S2 cont’d
E
F
G
H
I
J
K
L
