# Supplementary material for: Inhibiting the glycerophosphodiesterase EDI3 in ER-HER2+ breast cancer cells resistant to HER2-targeted therapy reduces viability and tumour growth
Source: J Exp Clin Cancer Res. 2023 Jan 20;42:25. doi: 10.1186/s13046-022-02578-w (PMC9854078; doi:10.1186/s13046-022-02578-w)
Supplement: Supplementary file 3 — Additional file 3: Supplementary Figure S3. Effect of targeting EDI3 on viability, alone or in combination with lapatinib, in ER-HER2+ and ER+HER2+ breast cancer cell lines. A-B, EDI3 mRNA (left panels) and protein expression (right panels) after silencing EDI3 using siRNA in A, BT474 and B, EFM192A cells. C, Effect of inhibiting HER2 using increasing concentrations of lapatinib on viability in SKBR3, HCC1954, BT474 and EFM192A cells. D-G Influence of silencing EDI3 with siRNA (oligo #2) and inhibiting HER2 with lapatinib (0.01 µM, 0.1 µM and 1 µM), as well as the combined inhibition of both EDI3 and HER2 on viability in D, SKBR3, E, HCC1954, F, BT474 and G, EFM192A cells. H, Effect of inhibiting HER2 using different concentrations of trastuzumab on viability in SKBR3, HCC1954, BT474 and EFM192A cells. I-L Influence of inhibiting EDI3 with siRNA (oligos #1 and #2) and HER2 with trastuzumab (1 µg/ml and 10 µg/ml), as well as the combined inhibition of both EDI3 and HER2 on viability in I, SKBR3, J, HCC1954, K, BT474 and L, EFM192A. Data are mean ± SD (A-C and H) or mean ± SE (D-G and I-L) of at least three independent experiments (*, P <0.05; **, P < 0.01; ***, P < 0.001; ****P < 0.0001; ns, not significant). [file 13046_2022_2578_MOESM3_ESM.pptx]

## Slide 1
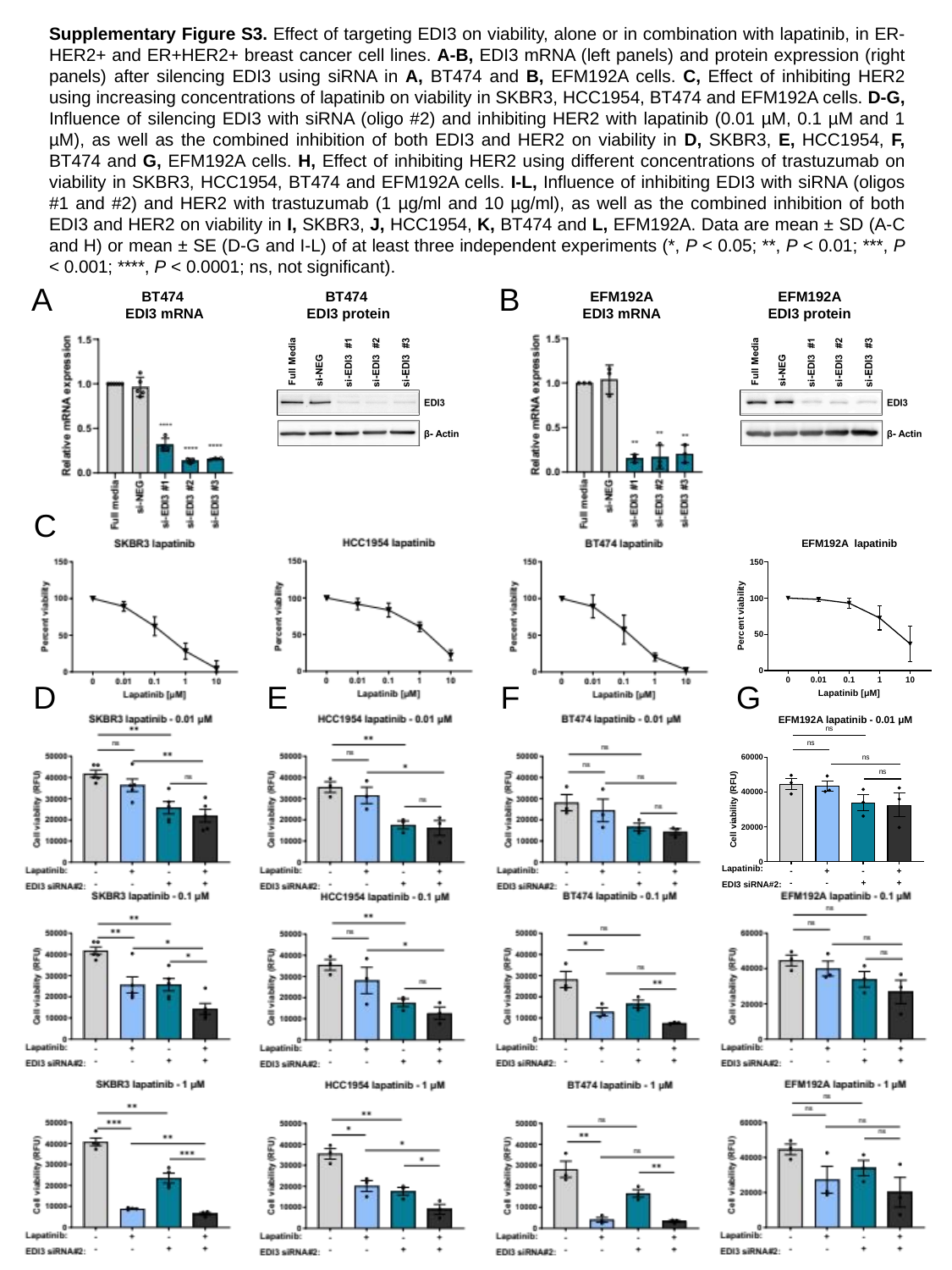

Supplementary Figure S3. Effect of targeting EDI3 on viability, alone or in combination with lapatinib, in ER-HER2+ and ER+HER2+ breast cancer cell lines. A-B, EDI3 mRNA (left panels) and protein expression (right panels) after silencing EDI3 using siRNA in A, BT474 and B, EFM192A cells. C, Effect of inhibiting HER2 using increasing concentrations of lapatinib on viability in SKBR3, HCC1954, BT474 and EFM192A cells. D-G, Influence of silencing EDI3 with siRNA (oligo #2) and inhibiting HER2 with lapatinib (0.01 µM, 0.1 µM and 1 µM), as well as the combined inhibition of both EDI3 and HER2 on viability in D, SKBR3, E, HCC1954, F, BT474 and G, EFM192A cells. H, Effect of inhibiting HER2 using different concentrations of trastuzumab on viability in SKBR3, HCC1954, BT474 and EFM192A cells. I-L, Influence of inhibiting EDI3 with siRNA (oligos #1 and #2) and HER2 with trastuzumab (1 µg/ml and 10 µg/ml), as well as the combined inhibition of both EDI3 and HER2 on viability in I, SKBR3, J, HCC1954, K, BT474 and L, EFM192A. Data are mean ± SD (A-C and H) or mean ± SE (D-G and I-L) of at least three independent experiments (*, P < 0.05; **, P < 0.01; ***, P < 0.001; ****, P < 0.0001; ns, not significant).
A
B
BT474
EDI3 mRNA
BT474
EDI3 protein
EFM192A
EDI3 mRNA
EFM192A
EDI3 protein
C
D
E
F
G

## Slide 2
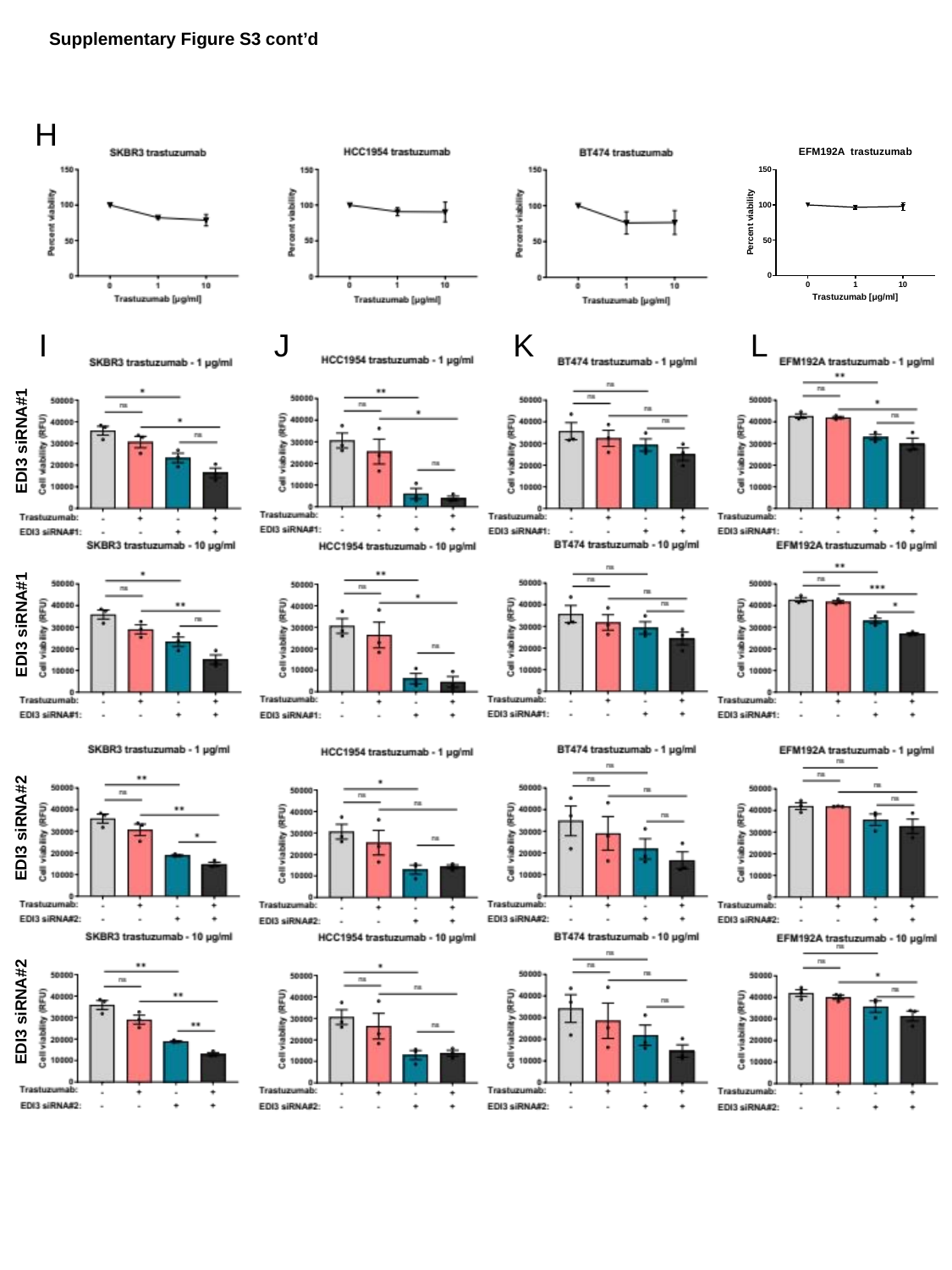

Supplementary Figure S3 cont’d
H
I
J
K
L
EDI3 siRNA#1
EDI3 siRNA#1
EDI3 siRNA#2
EDI3 siRNA#2
