# Supplementary material for: Inhibiting the glycerophosphodiesterase EDI3 in ER-HER2+ breast cancer cells resistant to HER2-targeted therapy reduces viability and tumour growth
Source: J Exp Clin Cancer Res. 2023 Jan 20;42:25. doi: 10.1186/s13046-022-02578-w (PMC9854078; doi:10.1186/s13046-022-02578-w)
Supplement: Supplementary file 4 — Additional file 4: Supplementary Figure S4. Inhibiting EDI3 with dipyridamole, alone or in combination with HER2-targeting therapy, in ER-HER2+ tumors in mice. A-D EDI3 activity in percent after treatment with dipyridamole at different concentrations in A, HCC1954 B, SKBR3, C, BT474 and D, EFM192 cells. E, Cell viability in percent of vehicle control after treatment with different concentrations of dipyridamole in SKBR3, HCC1954, BT474 and EFM192A cells (upper panel) and EC50-values with corresponding 95% confidence intervals (CI) (lower panel). F, Concentration of dipyridamole in plasma after a single oral dose of 120 mg/kg dipyridamole in mice over time. G, Mouse weight (grams) after treatment with dipyridamole, lapatinib and the combination for up to 4 weeks. H, Images of CD1 nude mice with tumors encircled, after 4-week treatment of vehicle, dipyridamole, lapatinib and the combination of both. I, Plasma and tumor concentrations (µM) of dipyridamole (upper panel) and lapatinib (lower panel) after treatment with each compound alone or in combination (D+L). Data (A-E) are mean ± SD of three independent experiments; mean ± SD of three CD1 mice (F); mean ± SD 5 to 6 mice (G); and representative images of 4 mice per condition H. D+L (combined dipyridamole and lapatinib treatment); BQL (below quantification limit); EC50 (half maximal effective concentration). [file 13046_2022_2578_MOESM4_ESM.pptx]

## Slide 1
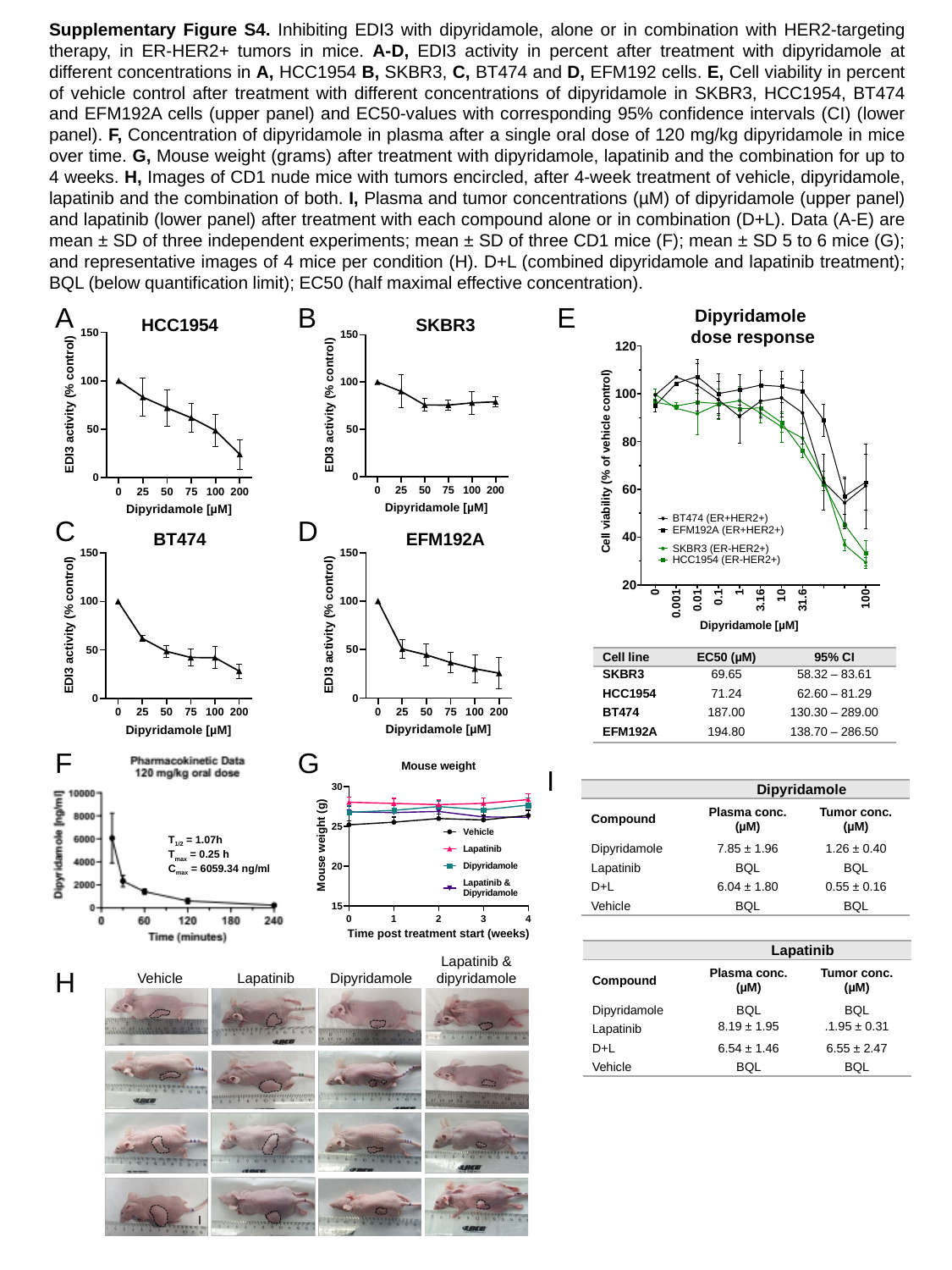

Supplementary Figure S4. Inhibiting EDI3 with dipyridamole, alone or in combination with HER2-targeting therapy, in ER-HER2+ tumors in mice. A-D, EDI3 activity in percent after treatment with dipyridamole at different concentrations in A, HCC1954 B, SKBR3, C, BT474 and D, EFM192 cells. E, Cell viability in percent of vehicle control after treatment with different concentrations of dipyridamole in SKBR3, HCC1954, BT474 and EFM192A cells (upper panel) and EC50-values with corresponding 95% confidence intervals (CI) (lower panel). F, Concentration of dipyridamole in plasma after a single oral dose of 120 mg/kg dipyridamole in mice over time. G, Mouse weight (grams) after treatment with dipyridamole, lapatinib and the combination for up to 4 weeks. H, Images of CD1 nude mice with tumors encircled, after 4-week treatment of vehicle, dipyridamole, lapatinib and the combination of both. I, Plasma and tumor concentrations (µM) of dipyridamole (upper panel) and lapatinib (lower panel) after treatment with each compound alone or in combination (D+L). Data (A-E) are mean ± SD of three independent experiments; mean ± SD of three CD1 mice (F); mean ± SD 5 to 6 mice (G); and representative images of 4 mice per condition (H). D+L (combined dipyridamole and lapatinib treatment); BQL (below quantification limit); EC50 (half maximal effective concentration).
A
B
E
Dipyridamole
dose response
HCC1954
SKBR3
C
D
BT474
EFM192A
| Cell line | EC50 (µM) | 95% CI |
| --- | --- | --- |
| SKBR3 | 69.65 | 58.32 – 83.61 |
| HCC1954 | 71.24 | 62.60 – 81.29 |
| BT474 | 187.00 | 130.30 – 289.00 |
| EFM192A | 194.80 | 138.70 – 286.50 |
F
G
I
| | Dipyridamole | |
| --- | --- | --- |
| Compound | Plasma conc. (µM) | Tumor conc. (µM) |
| Dipyridamole | 7.85 ± 1.96 | 1.26 ± 0.40 |
| Lapatinib | BQL | BQL |
| D+L | 6.04 ± 1.80 | 0.55 ± 0.16 |
| Vehicle | BQL | BQL |
T1/2 = 1.07h
Tmax = 0.25 h
Cmax = 6059.34 ng/ml
| | Lapatinib | |
| --- | --- | --- |
| Compound | Plasma conc. (µM) | Tumor conc. (µM) |
| Dipyridamole | BQL | BQL |
| Lapatinib | 8.19 ± 1.95 | .1.95 ± 0.31 |
| D+L | 6.54 ± 1.46 | 6.55 ± 2.47 |
| Vehicle | BQL | BQL |
Lapatinib &
dipyridamole
H
Vehicle
Lapatinib
Dipyridamole
