# Supplementary material for: Inhibiting the glycerophosphodiesterase EDI3 in ER-HER2+ breast cancer cells resistant to HER2-targeted therapy reduces viability and tumour growth
Source: J Exp Clin Cancer Res. 2023 Jan 20;42:25. doi: 10.1186/s13046-022-02578-w (PMC9854078; doi:10.1186/s13046-022-02578-w)
Supplement: Supplementary file 5 — Additional file 5: Supplemental Figure S5. Inducibly silencing EDI3 inhibits colony formation and cell viability. A, Representative images (top) and corresponding quantification (bottom) of colony number (left) and size (right) formed by HCC1954 shNEG and HCC1954 shEDI3 (oligos shEDI3 #1, #2, #3) cells treated with 0 or 0.1 µg/ml doxycycline. B, Viability (RFU) after treating HCC1954 shNEG and HCC1954 shEDI3 (oligos shEDI3 #1, #2) with 0.1 or 1 µg/ml doxycycline. All in vitro data are mean ± SD of three independent experiments. (*, P < 0.05; **, P < 0.01; ***, P < 0.001). RFU, relative fluorescence units. [file 13046_2022_2578_MOESM5_ESM.pptx]

## Slide 1
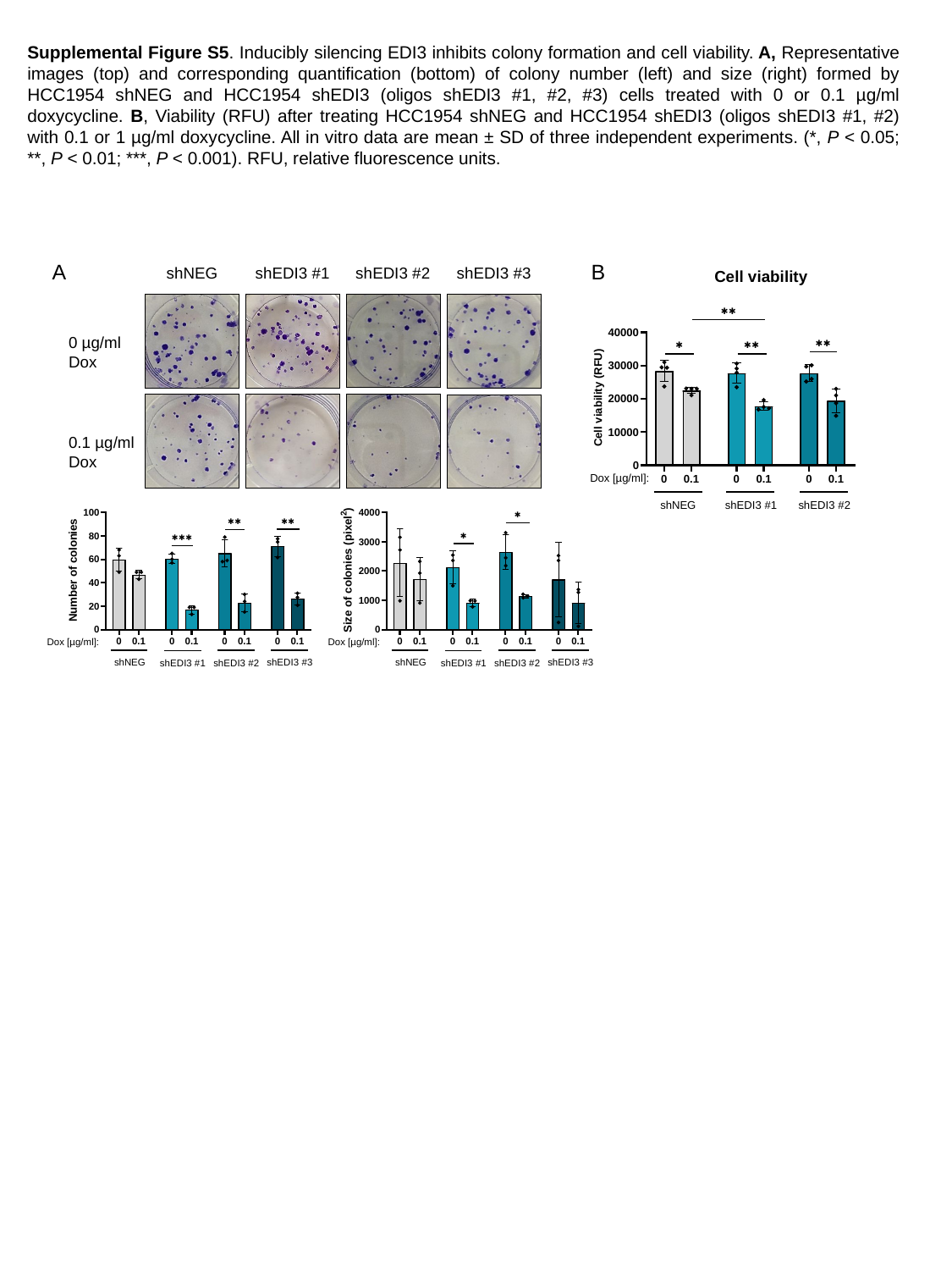

Supplemental Figure S5. Inducibly silencing EDI3 inhibits colony formation and cell viability. A, Representative images (top) and corresponding quantification (bottom) of colony number (left) and size (right) formed by HCC1954 shNEG and HCC1954 shEDI3 (oligos shEDI3 #1, #2, #3) cells treated with 0 or 0.1 µg/ml doxycycline. B, Viability (RFU) after treating HCC1954 shNEG and HCC1954 shEDI3 (oligos shEDI3 #1, #2) with 0.1 or 1 µg/ml doxycycline. All in vitro data are mean ± SD of three independent experiments. (*, P < 0.05; **, P < 0.01; ***, P < 0.001). RFU, relative fluorescence units.
A
B
shNEG
shEDI3 #1
shEDI3 #2
shEDI3 #3
Cell viability
0 µg/ml Dox
0.1 µg/ml Dox
