# Supplementary material for: Inhibiting the glycerophosphodiesterase EDI3 in ER-HER2+ breast cancer cells resistant to HER2-targeted therapy reduces viability and tumour growth
Source: J Exp Clin Cancer Res. 2023 Jan 20;42:25. doi: 10.1186/s13046-022-02578-w (PMC9854078; doi:10.1186/s13046-022-02578-w)
Supplement: Supplementary file 6 — Additional file 6: Supplementary Table S1. EDI3 (GPCPD1) expression in human breast cancer tissue was evaluated in publicly available Affymetrix HG U133 Plus 2.0 gene expression microarray dataset that were downloaded from the Gene Expression Omnibus webportal (GEO) together with available clinicopathological data. [file 13046_2022_2578_MOESM6_ESM.docx]

**Supplementary Table S1.** EDI3 (*GPCPD1*) expression in human breast cancer tissue was evaluated in publicly available Affymetrix HG U133 Plus 2.0 gene expression microarray dataset that were downloaded from the Gene Expression Omnibus webportal (GEO) together with available clinicopathological data.

|  | **Dataset (GEO accession number)** | | | | | |  |
| --- | --- | --- | --- | --- | --- | --- | --- |
|  | **GSE16446** | **GSE19615** | **GSE28844** | **GSE32646** | **GSE6532** | **GSE6532** | **Combined** |
| **Total** | 114 | 115 | 32 | 115 | 87 | 77 | 540 |
| **Age** |  |  |  |  |  |  |  |
| <50 years | 69 | 54 | 14 | 57 | 5 | 6 | 205 |
| ≥50 years | 45 | 61 | 18 | 58 | 82 | 71 | 335 |
| Missing | 0 | 0 | 0 | 0 | 0 | 0 | 0 |
| **Tumor size** |  |  |  |  |  |  |  |
| <2 cm | 0 | 50 | 0 | 0 | 30 | 28 | 108 |
| ≥2 cm | 0 | 65 | 0 | 0 | 57 | 49 | 171 |
| Missing | 114 | 0 | 32 | 115 | 0 | 0 | 261 |
| **Tumor grade** |  |  |  |  |  |  |  |
| Low (I+II) | 22 | 51 | 0 | 94 | 54 | 34 | 255 |
| High (III) | 87 | 64 | 0 | 21 | 16 | 24 | 212 |
| Missing | 5 | 0 | 32 | 0 | 17 | 19 | 73 |
| **ER status** |  |  |  |  |  |  |  |
| Negative | 114 | 51 | 9 | 54 | 2 | 3 | 233 |
| Positive | 0 | 64 | 23 | 61 | 85 | 74 | 307 |
| Missing | 0 | 0 | 0 | 0 | 0 | 0 | 0 |
| **HER2 status** |  |  |  |  |  |  |  |
| Negative | 90 | 97 | 25 | 98 | 81 | 72 | 463 |
| Positive | 24 | 18 | 7 | 17 | 6 | 5 | 77 |
| Missing | 0 | 0 | 0 | 0 | 0 | 0 | 0 |
| **Metastasis** |  |  |  |  |  |  |  |
| Yes | 25 | 14 | 0 | 0 | 28 | 10 | 77 |
| No | 89 | 101 | 0 | 0 | 59 | 67 | 316 |
| Missing | 0 | 0 | 32 | 115 | 0 | 0 | 147 |
