# Supplementary material for: Inhibiting the glycerophosphodiesterase EDI3 in ER-HER2+ breast cancer cells resistant to HER2-targeted therapy reduces viability and tumour growth
Source: J Exp Clin Cancer Res. 2023 Jan 20;42:25. doi: 10.1186/s13046-022-02578-w (PMC9854078; doi:10.1186/s13046-022-02578-w)
Supplement: Supplementary file 7 — Additional file 7: Supplementary Table S2. List of used reagents including A, QuantiTect primer assays, B, siRNA and shRNA oligos, and C, antibodies. [file 13046_2022_2578_MOESM7_ESM.docx]

**Supplementary Table S2.** List of used reagents including **A,** QuantiTect primer assays, **B,** siRNA and shRNA oligos, and **C,** antibodies.

|  | | | | |
| --- | --- | --- | --- | --- |
| **A QuantiTect primer assays** | | | | |
| **Gene** | **Primer assay** | **Article number** | | |
| GPCPD1 (EDI3) | Hs_GPCPD1_1_SG | QT00066598 | | |
| HER2 (ERBB2) | Hs_ERBB2_1_SG | QT00060746 | | |
| HER2 (ERBB2) | Rn_Erbb2_1_SG | QT00193466 | | |
| ACTB | Hs_ACTB_1_SG | QT00095431 | | |
| RPL37A | Hs_RPL37A_1_SG | QT00051758 | | |
|  | | | | |
| **B List of antibodies used** | | | | |
| **Protein target** | **Company** | **Article number** | | **Dilution** |
| EDI3, clone 3B8G3 | AMS Bio | Custom Made | | 1:1000 |
| EDI3, clone 4G7F3 | AMS Bio | Custom Made | | 1:500 |
| phospho-HER2 | Cell Signaling | 6942 | | 1:1000 |
| HER2 | Abcam | ab134182/EP1045Y | | 1:10,000 |
| phospho-mTOR | Cell Signaling | 2971S | | 1:1000 |
| mTOR | Cell Signaling | 2972S | | 1:1000 |
| phospho-GSK3β | Cell Signaling | 9323S | | 1:1000 |
| GSK3β | Cell Signaling | 9315S | | 1:1000 |
| β-catenin | Cell Signaling | 8480S | | 1:1000 |
| phospho-p44/42 MAPK | Cell Signaling | 4370S | | 1:2000 |
| p44/42 MAPK (ERK1/2) | Cell Signaling | 9102L | | 1:1000 |
| phospho-Akt | Cell Signaling | 4060S | | 1:1000 |
| Akt | Cell Signaling | 9272S | | 1:1000 |
| phospho-PKCα/βII | Cell Signaling | 9375S | | 1:1000 |
| PKCα | Cell Signaling | 2056S | | 1:1000 |
| β-actin (anti rabbit) | Cell Signaling | 4967S | | 1:1000 |
| β-actin (anti mouse) | Sigma | A5316 | | 1:3000 |
| Calnexin | Cell Signaling | 2433S | | 1:1000 |
| Anti-Rabbit IgG | Cell Signaling | 7074S | | 1:1000 |
| Anti-Mouse IgG | Cell Signaling | 7076S | | 1:1000 |
|  | | | | |
| **C List of oligos (siRNA oligos from Thermo Scientific; shRNA oligos from Dharmacon)** | | | | |
| **Gene** | **Product** | **Article number** | **siRNA sequence** | |
| si-EDI3 1 | Stealth RNAi™ | 1299001-HSS125509 | GGCAAAGUGAGAGUUGACUAUAUAA | |
| si-EDI3 2 | Silencer® Select | 4392420-s32105 | GGAUGGUAACUUAUCAACAtt | |
| si-EDI3 3 | Stealth RNAi™ | 1299001-HSS125510 | GCUCACUCAUGUGACUGCACUGAAA | |
| si-HER2 1 (si-ERBB2) | Stealth siRNA | 1299001- HSS103333 | GGUCACCUACAACACAGACACGUUU | |
| si-HER2 2 (si-ERBB2) | Stealth siRNA | 1299001- HSS103334 | GAGAUCACAGGUUACCUAUACAUCU | |
| si-HER2 3 (si-ERBB2) | Stealth siRNA | 1299001- HSS103335 | GCCCAGCCUUCGACAACCUCUAUUA | |
| Negative Control Lo GC Duplex #2 | Stealth RNAi™ | 12935-110 | Not provided | |
| Negative Control No. 1 | Silencer® Select | 4390844 | Not provided | |
| shEDI3 #1 | SMARTvector^TM^ | V3SH7669-228754853 | CCAGAAGATGTAGGGTTTA | |
| shEDI3 #2 | SMARTvector^TM^ | V3SH7669-230425247 | GGAGTTAATGGTCTAATTT | |
| shEDI3 #3 | SMARTvector^TM^ | V3SH7669-225840029 | GGTACAGCTTGTCTCTTAT | |
| shNEG | SMARTvector^TM^ | VSC6571 | Not provided | |
