# Supplementary material for: Inhibiting the glycerophosphodiesterase EDI3 in ER-HER2+ breast cancer cells resistant to HER2-targeted therapy reduces viability and tumour growth
Source: J Exp Clin Cancer Res. 2023 Jan 20;42:25. doi: 10.1186/s13046-022-02578-w (PMC9854078; doi:10.1186/s13046-022-02578-w)
Supplement: Supplementary file 8 — Additional file 8: Supplementary Table S3. Association of EDI3 expression for the three probesets on the Affymetrix HG U133 Plus 2.0 array and available clinicopathological parameters shown for A, the six datasets combined, as well as separately for B, GSE16446, C, GSE19615, D, GSE28844, E, GSE32646, F, GSE6532, and G, GSE9195. P value from the Mann Whitney or Kruskal-Wallis U test. [file 13046_2022_2578_MOESM8_ESM.docx]

**Supplementary Table S3.** Association of EDI3 expression for the three probesets on the Affymetrix HG U133 Plus 2.0 array and available clinicopathological parameters shown for **A,** the six datasets combined, as well as separately for **B,** GSE16446, **C,** GSE19615, **D,** GSE28844, **E,** GSE32646, **F,** GSE6532, and **G,** GSE9195. *P* value from the Mann Whitney U or Kruskal-Wallis test.

|  |  |  | EDI3 probeset (HG U133 Plus 2.0) | | |
| --- | --- | --- | --- | --- | --- |
| A Combined |  |  | **224826_at** | **224835_at** | **230492_s_at** |
|  | **N** | **%** | **P** | **P** | **P** |
| Total | 540 | 100.0 |  |  |  |
| Age |  |  |  |  |  |
| <50 years | 205 | 38.0 | 0.926 | 0.779 | <0.001 |
| ≥50 years | 335 | 62.0 |  |  |  |
| Missing | 0 | 0.0 |  |  |  |
| Tumor size |  |  |  |  |  |
| <2 cm | 108 | 20.0 | 0.689 | 0.240 | 0.981 |
| ≥2 cm | 171 | 31.7 |  |  |  |
| Missing | 261 | 48.3 |  |  |  |
| Tumor grade |  |  |  |  |  |
| Low (I+II) | 255 | 47.2 | 0.021 | <0.001 | 0.009 |
| High (III) | 212 | 39.3 |  |  |  |
| Missing | 73 | 13.5 |  |  |  |
| ER status |  |  |  |  |  |
| Negative | 229 | 42.4 | 0.015 | 0.072 | <0.001 |
| Positive | 311 | 57.6 |  |  |  |
| Missing | 0 | 0.0 |  |  |  |
| HER2 status |  |  |  |  |  |
| Negative | 463 | 85.7 | <0.001 | 0.059 | <0.001 |
| Positive | 77 | 14.3 |  |  |  |
| Missing | 0 | 0.0 |  |  |  |
| Metastasis |  |  |  |  |  |
| Yes | 77 | 14.3 | 0.587 | 0.064 | 0.762 |
| No | 316 | 58.5 |  |  |  |
| Missing | 147 | 27.2 |  |  |  |

|  |  |  | EDI3 probeset (HG U133 Plus 2.0) | | |
| --- | --- | --- | --- | --- | --- |
| B GSE16446 |  |  | **224826_at** | **224835_at** | **230492_s_at** |
|  | **N** | **%** | **P** | **P** | **P** |
| Total | 114 | 100.0 |  |  |  |
| Age |  |  |  |  |  |
| <50 years | 69 | 60.5 | 0.176 | 0.864 | 0.739 |
| ≥50 years | 45 | 39.5 |  |  |  |
| Missing | 0 | 0.0 |  |  |  |
| Tumor size |  |  |  |  |  |
| <2 cm | 0 | 0.0 |  |  |  |
| ≥2 cm | 0 | 0.0 |  |  |  |
| Missing | 114 | 100.0 |  |  |  |
| Tumor grade |  |  |  |  |  |
| Low (I+II) | 22 | 19.3 | 0.046 | 0.334 | 0.135 |
| High (III) | 87 | 76.3 |  |  |  |
| Missing | 5 | 4.4 |  |  |  |
| ER status |  |  |  |  |  |
| Negative | 114 | 100.0 |  |  |  |
| Positive | 0 | 0.0 |  |  |  |
| Missing | 0 | 0.0 |  |  |  |
| HER2 status |  |  |  |  |  |
| Negative | 90 | 78.9 | <0.001 | 0.110 | <0.001 |
| Positive | 24 | 21.1 |  |  |  |
| Missing | 0 | 0.0 |  |  |  |
| Metastasis |  |  |  |  |  |
| Yes | 25 | 21.9 | 0.398 | 0.600 | 0.368 |
| No | 89 | 78.1 |  |  |  |
| Missing | 0 | 0.0 |  |  |  |


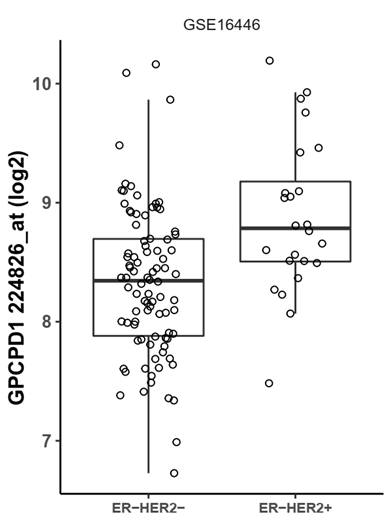
 P = 0.000454 (Mann-Whitney U)

|  |  |  | EDI3 probeset (HG U133 Plus 2.0) | | |
| --- | --- | --- | --- | --- | --- |
| C GSE19615 |  |  | **224826_at** | **224835_at** | **230492_s_at** |
|  | **N** | **%** | **P** | **P** | **P** |
| Total | 115 | 100.0 |  |  |  |
| Age |  |  |  |  |  |
| <50 years | 54 | 47.0 | 0.893 | 0.327 | 0.179 |
| ≥50 years | 61 | 53.0 |  |  |  |
| Missing | 0 | 0.0 |  |  |  |
| Tumor size |  |  |  |  |  |
| <2 cm | 50 | 43.5 | 0.580 | 0.119 | 0.874 |
| ≥2 cm | 65 | 56.5 |  |  |  |
| Missing | 0 | 0.0 |  |  |  |
| Tumor grade |  |  |  |  |  |
| Low (I+II) | 51 | 44.3 | 0.197 | <0.001 | 0.122 |
| High (III) | 64 | 55.7 |  |  |  |
| Missing | 0 | 0.0 |  |  |  |
| ER status |  |  |  |  |  |
| Negative | 51 | 44.3 | 0.593 | 0.002 | 0.003 |
| Positive | 64 | 55.7 |  |  |  |
| Missing | 0 | 0.0 |  |  |  |
| HER2 status |  |  |  |  |  |
| Negative | 97 | 84.3 | 0.003 | 0.271 | <0.001 |
| Positive | 18 | 15.7 |  |  |  |
| Missing | 0 | 0.0 |  |  |  |
| Metastasis |  |  |  |  |  |
| Yes | 14 | 12.2 | 0.238 | 0.182 | 0.871 |
| No | 101 | 87.8 |  |  |  |
| Missing | 0 | 0.0 |  |  |  |


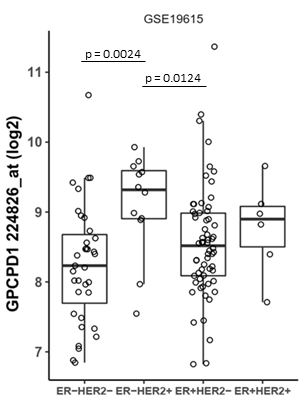
 P = 0.007076 (Kruskal-Wallis)

|  |  |  | EDI3 probeset (HG U133 Plus 2.0) | | |
| --- | --- | --- | --- | --- | --- |
| D GSE28844 |  |  | **224826_at** | **224835_at** | **230492_s_at** |
|  | **N** | **%** | **P** | **P** | **P** |
| Total | 32 | 100.0 |  |  |  |
| Age |  |  |  |  |  |
| <50 years | 14 | 43.8 | 0.020 | 0.037 | 0.006 |
| ≥50 years | 18 | 56.3 |  |  |  |
| Missing | 0 | 0.0 |  |  |  |
| Tumor size |  |  |  |  |  |
| <2 cm | 0 | 0.0 |  |  |  |
| ≥2 cm | 0 | 0.0 |  |  |  |
| Missing | 32 | 100.0 |  |  |  |
| Tumor grade |  |  |  |  |  |
| Low (I+II) | 0 | 0.0 |  |  |  |
| High (III) | 0 | 0.0 |  |  |  |
| Missing | 32 | 100.0 |  |  |  |
| ER status |  |  |  |  |  |
| Negative | 9 | 28.1 | 0.001 | 0.246 | 0.001 |
| Positive | 23 | 71.9 |  |  |  |
| Missing | 0 | 0.0 |  |  |  |
| HER2 status |  |  |  |  |  |
| Negative | 25 | 78.1 | 0.007 | 0.068 | 0.003 |
| Positive | 7 | 21.9 |  |  |  |
| Missing | 0 | 0.0 |  |  |  |
| Metastasis |  |  |  |  |  |
| Yes | 0 | 0.0 |  |  |  |
| No | 0 | 0.0 |  |  |  |
| Missing | 32 | 100.0 |  |  |  |


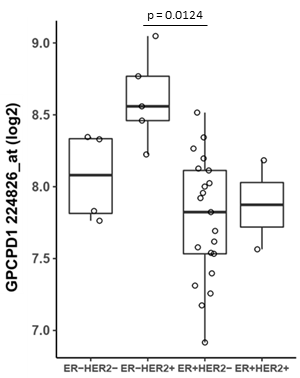
 P = 0.008446 (Kruskal-Wallis)

|  |  |  | EDI3 probeset (HG U133 Plus 2.0) | | |
| --- | --- | --- | --- | --- | --- |
| E GSE32646 |  |  | **224826_at** | **224835_at** | **230492_s_at** |
|  | **N** | **%** | **P** | **P** | **P** |
| Total | 115 | 100.0 |  |  |  |
| Age |  |  |  |  |  |
| <50 years | 57 | 49.6 | 0.275 | 0.611 | 0.146 |
| ≥50 years | 58 | 50.4 |  |  |  |
| Missing | 0 | 0.0 |  |  |  |
| Tumor size |  |  |  |  |  |
| <2 cm | 0 | 0.0 |  |  |  |
| ≥2 cm | 0 | 0.0 |  |  |  |
| Missing | 115 | 100.0 |  |  |  |
| Tumor grade |  |  |  |  |  |
| Low (I+II) | 94 | 81.7 | 0.828 | 0.977 | 0.332 |
| High (III) | 21 | 18.3 |  |  |  |
| Missing | 0 | 0.0 |  |  |  |
| ER status |  |  |  |  |  |
| Negative | 54 | 47.0 | 0.049 | 0.012 | 0.004 |
| Positive | 61 | 53.0 |  |  |  |
| Missing | 0 | 0.0 |  |  |  |
| HER2 status |  |  |  |  |  |
| Negative | 98 | 85.2 | 0.004 | 0.273 | 0.004 |
| Positive | 17 | 14.8 |  |  |  |
| Missing | 0 | 0.0 |  |  |  |
| Metastasis |  |  |  |  |  |
| Yes | 0 | 0.0 |  |  |  |
| No | 0 | 0.0 |  |  |  |
| Missing | 115 | 100.0 |  |  |  |


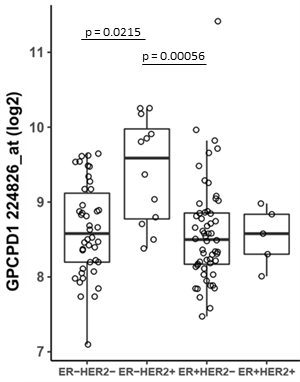
 P = 0.00498 (Kruskal-Wallis)

|  |  |  | EDI3 probeset (HG U133 Plus 2.0) | | |
| --- | --- | --- | --- | --- | --- |
| F GSE6532 |  |  | **224826_at** | **224835_at** | **230492_s_at** |
|  | **N** | **%** | **P** | **P** | **P** |
| Total | 87 | 100.0 |  |  |  |
| Age |  |  |  |  |  |
| <50 years | 5 | 5.7 | 0.511 | 0.827 | 0.466 |
| ≥50 years | 82 | 94.3 |  |  |  |
| Missing | 0 | 0.0 |  |  |  |
| Tumor size |  |  |  |  |  |
| <2 cm | 30 | 34.5 | 0.809 | 0.605 | 0.192 |
| ≥2 cm | 57 | 65.5 |  |  |  |
| Missing | 0 | 0.0 |  |  |  |
| Tumor grade |  |  |  |  |  |
| Low (I+II) | 54 | 62.1 | 0.467 | 0.484 | 0.121 |
| High (III) | 16 | 18.4 |  |  |  |
| Missing | 17 | 19.5 |  |  |  |
| ER status |  |  |  |  |  |
| Negative | 2 | 2.3 | 0.083 | 0.481 | 0.019 |
| Positive | 85 | 97.7 |  |  |  |
| Missing | 0 | 0.0 |  |  |  |
| HER2 status |  |  |  |  |  |
| Negative | 81 | 93.1 | 0.159 | 0.592 | 0.056 |
| Positive | 6 | 6.9 |  |  |  |
| Missing | 0 | 0.0 |  |  |  |
| Metastasis |  |  |  |  |  |
| Yes | 28 | 32.2 | 0.669 | 0.345 | 0.764 |
| No | 59 | 67.8 |  |  |  |
| Missing | 0 | 0.0 |  |  |  |


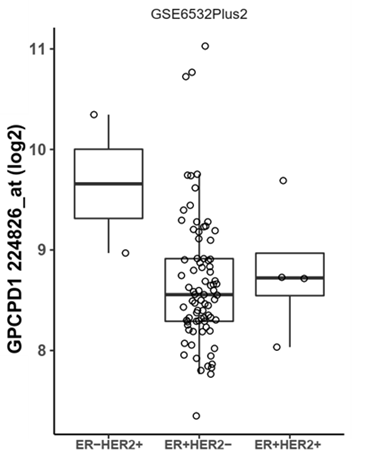
 P = 0.1884 (Kruskal-Wallis)

|  |  |  | EDI3 probeset (HG U133 Plus 2.0) | | |
| --- | --- | --- | --- | --- | --- |
| G GSE9195 |  |  | **224826_at** | **224835_at** | **230492_s_at** |
|  | **N** | **%** | **P** | **P** | **P** |
| Total | 77 | 100.0 |  |  |  |
| Age |  |  |  |  |  |
| <50 years | 6 | 7.8 | 0.955 | 0.718 | 0.718 |
| ≥50 years | 71 | 92.2 |  |  |  |
| Missing | 0 | 0.0 |  |  |  |
| Tumor size |  |  |  |  |  |
| <2 cm | 28 | 36.4 | 1.000 | 0.824 | 0.379 |
| ≥2 cm | 49 | 63.6 |  |  |  |
| Missing | 0 | 0.0 |  |  |  |
| Tumor grade |  |  |  |  |  |
| Low (I+II) | 34 | 44.2 | 0.987 | 0.528 | 0.236 |
| High (III) | 24 | 31.2 |  |  |  |
| Missing | 19 | 24.7 |  |  |  |
| ER status |  |  |  |  |  |
| Negative | 3 | 3.9 | 0.003 | 0.017 | 0.031 |
| Positive | 74 | 96.1 |  |  |  |
| Missing | 0 | 0.0 |  |  |  |
| HER2 status |  |  |  |  |  |
| Negative | 72 | 93.5 | 0.306 | 0.896 | 0.251 |
| Positive | 5 | 6.5 |  |  |  |
| Missing | 0 | 0.0 |  |  |  |
| Metastasis |  |  |  |  |  |
| Yes | 10 | 13.0 | 0.544 | 0.682 | 0.379 |
| No | 67 | 87.0 |  |  |  |
| Missing | 0 | 0.0 |  |  |  |


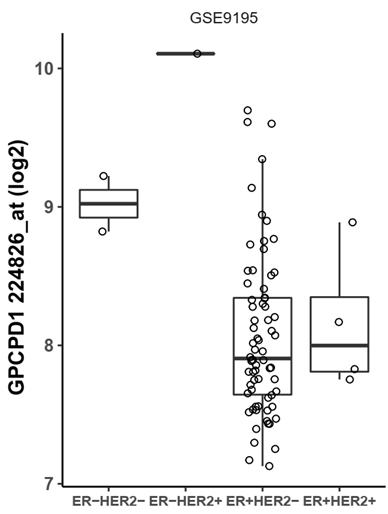
 P = 0.0704 (Kruskal-Wallis)
